# Changing genetic architecture of body mass index from infancy to early adulthood: an individual based pooled analysis of 25 twin cohorts

**DOI:** 10.1038/s41366-022-01202-3

**Published:** 2022-08-09

**Authors:** Karri Silventoinen, Weilong Li, Aline Jelenkovic, Reijo Sund, Yoshie Yokoyama, Sari Aaltonen, Maarit Piirtola, Masumi Sugawara, Mami Tanaka, Satoko Matsumoto, Laura A. Baker, Catherine Tuvblad, Per Tynelius, Finn Rasmussen, Jeffrey M. Craig, Richard Saffery, Gonneke Willemsen, Meike Bartels, Catharina E. M. van Beijsterveldt, Nicholas G. Martin, Sarah E. Medland, Grant W. Montgomery, Paul Lichtenstein, Robert F. Krueger, Matt McGue, Shandell Pahlen, Kaare Christensen, Axel Skytthe, Kirsten O. Kyvik, Kimberly J. Saudino, Lise Dubois, Michel Boivin, Mara Brendgen, Ginette Dionne, Frank Vitaro, Vilhelmina Ullemar, Catarina Almqvist, Patrik K. E. Magnusson, Robin P. Corley, Brooke M. Huibregtse, Ariel Knafo-Noam, David Mankuta, Lior Abramson, Claire M. A. Haworth, Robert Plomin, Morten Bjerregaard-Andersen, Henning Beck-Nielsen, Morten Sodemann, Glen E. Duncan, Dedra Buchwald, S. Alexandra Burt, Kelly L. Klump, Clare H. Llewellyn, Abigail Fisher, Dorret I. Boomsma, Thorkild I. A. Sørensen, Jaakko Kaprio

**Affiliations:** 1 Population Research Unit, Faculty of Social Sciences, University of Helsinki, Helsinki, Finland; 2 Center for Twin Research, Osaka University Graduate School of Medicine, Osaka, Japan; 3 Department of Physiology, Faculty of Medicine and Nursing, University of the Basque Country, Leioa, Spain; 4 Department of Public Health, University of Helsinki, Helsinki, Finland; 5 Institute of Clinical Medicine, University of Eastern Finland, Kuopio, Finland; 6 Department of Public Health Nursing, Osaka Metropolitan University, Osaka, Japan; 7 Institute for Molecular Medicine Finland FIMM, Helsinki, Finland; 8 UKK Institute – Centre for Health Promotion Research, Tampere, Finland; 9 Faculty of Human Studies, Shirayuri University, Tokyo, Japan; 10 Center for Forensic Mental Health, Chiba University, Chiba, Japan; 11 Institute for Education and Human Development, Ochanomizu University, Tokyo, Japan; 12 Department of Psychology, University of Southern California, Los Angeles, CA USA; 13 School of Law, Psychology and Social Work, Örebro University, Örebro, Sweden; 14 Department of Global Public Health, Karolinska Institutet, Stockholm, Sweden; 15 The Institute for Mental and Physical Health and Clinical Translation (IMPACT), Deakin University School of Medicine, Geelong, Australia; 16 Murdoch Childrens Research Institute, Royal Children’s Hospital, Parkville, Victoria Australia; 17 Department of Paediatrics, University of Melbourne, Parkville, Victoria Australia; 18 Netherlands Twin Register, Department of Biological Psychology, Vrije Universiteit, Amsterdam, Amsterdam, Netherlands; 19 Genetic Epidemiology Department, QIMR Berghofer Medical Research Institute, Brisbane, Australia; 20 Institute for Molecular Bioscience, The University of Queensland, Brisbane, Australia; 21 Department of Medical Epidemiology and Biostatistics, Karolinska Institutet, Stockholm, Sweden; 22 Department of Psychology, University of Minnesota, Minneapolis, MN USA; 23 Department of Psychology, University of California, Riverside, Riverside, CA 92521 USA; 24 The Danish Twin Registry, Department of Public Health, Epidemiology, Biostatistics & Biodemography, University of Southern Denmark Odense, Odense, Denmark; 25 Department of Clinical Biochemistry and Pharmacology and Department of Clinical Genetics, Odense University Hospital, Odense, Denmark; 26 Department of Clinical Research, University of Southern Denmark, Odense, Denmark; 27 Odense Patient data Explorative Network (OPEN), Odense University Hospital, Odense, Denmark; 28 Boston University, Department of Psychological and Brain Sciencies, Boston, MA USA; 29 School of Epidemiology and Public Health, University of Ottawa, Ottawa, Ontario, Canada; 30 École de psychologie, Université Laval, Québec, Canada; 31 Département de psychologie, Université du Québec à Montréal, Montréal, Québec, Canada; 32 École de psychoéducation, Université de Montréal, Montréal, Québec Canada; 33 Division of Obstetrics and Gynecology, Department of Clinical Science, Intervention and Technology (CLINTEC), Karolinska Institutet, Stockholm, Sweden; 34 Theme Women’s Health, Karolinska University Hospital, Karolinska University Hospital, Stockholm, Sweden; 35 Pediatric Allergy and Pulmonology Unit at Astrid Lindgren Children’s Hospital, Karolinska University Hospital, Stockholm, Sweden; 36 Institute for Behavioral Genetics, University of Colorado, Boulder, Colorado USA; 37 Department of Psychology and Neuroscience, University of Colorado, Boulder, Colorado USA; 38 The Hebrew University of Jerusalem, Jerusalem, Israel; 39 Hadassah Hospital Obstetrics and Gynecology Department, Hebrew University Medical School, Jerusalem, Israel; 40 School of Psychological Science, University of Bristol, Bristol, UK; 41 Social Genetic and Developmental Psychiatry Centre, Institute of Psychiatry, Psychology and Neuroscience, King’s College London, London, UK; 42 Bandim Health Project, INDEPTH Network, Bissau, Guinea-Bissau; 43 Department of Endocrinology, Hospital of Southwest Jutland, Esbjerg, Denmark; 44 Department of Endocrinology, Odense University Hospital, Odense, Denmark; 45 Department of Infectious Diseases, Odense University Hospital, Odense, Denmark; 46 Washington State Twin Registry, Washington State University - Health Sciences Spokane, Spokane, WA USA; 47 Department of Psychology, Michigan State University, East Lansing, Michigan USA; 48 Department of Behavioural Science and Health, Institute of Epidemiology and Health Care, University College London, London, UK; 49 Novo Nordisk Foundation Centre for Basic Metabolic Research, Faculty of Health and Medical Sciences, University of Copenhagen, Copenhagen, Denmark; 50 Department of Public Health (Section of Epidemiology), Faculty of Health and Medical Sciences, University of Copenhagen, Copenhagen, Denmark

**Keywords:** Risk factors, Epidemiology

## Abstract

**Background:**

Body mass index (BMI) shows strong continuity over childhood and adolescence and high childhood BMI is the strongest predictor of adult obesity. Genetic factors strongly contribute to this continuity, but it is still poorly known how their contribution changes over childhood and adolescence. Thus, we used the genetic twin design to estimate the genetic correlations of BMI from infancy to adulthood and compared them to the genetic correlations of height.

**Methods:**

We pooled individual level data from 25 longitudinal twin cohorts including 38,530 complete twin pairs and having 283,766 longitudinal height and weight measures. The data were analyzed using Cholesky decomposition offering genetic and environmental correlations of BMI and height between all age combinations from 1 to 19 years of age.

**Results:**

The genetic correlations of BMI and height were stronger than the trait correlations. For BMI, we found that genetic correlations decreased as the age between the assessments increased, a trend that was especially visible from early to middle childhood. In contrast, for height, the genetic correlations were strong between all ages. Age-to-age correlations between environmental factors shared by co-twins were found for BMI in early childhood but disappeared altogether by middle childhood. For height, shared environmental correlations persisted from infancy to adulthood.

**Conclusions:**

Our results suggest that the genes affecting BMI change over childhood and adolescence leading to decreasing age-to-age genetic correlations. This change is especially visible from early to middle childhood indicating that new genetic factors start to affect BMI in middle childhood. Identifying mediating pathways of these genetic factors can open possibilities for interventions, especially for those children with high genetic predisposition to adult obesity.

## Introduction

Obesity is one of the most important modifiable risk factors of health in the modern world [1]. Especially worrying is the global prevalence of adiposity in childhood and adolescence, which has rapidly increased during the last decades [2]. These trends leveled off in Western countries at a high level at the beginning of the 21^st^ century but continued in other parts of the world [3]. Childhood adiposity predisposes for health risks in adulthood [4, 5], and thus the high level of adiposity in childhood creates an underlying threat to global health. However, this epidemic may be preventable since high childhood body mass index (BMI) seems to affect adult diseases especially through adult BMI [6]. Previous studies have demonstrated that there is strong continuity of BMI over childhood [7], and that high childhood BMI is the most important risk factor for adult obesity [8]. Sustainable weight loss in adulthood is very difficult to obtain because of several psychological and physiological mechanisms preventing weight loss and promoting weigh regain; in contrast, excess body fat in childhood can be lost without weight loss as the child grows and develops [9]. Thus, effective interventions to slow or even eliminate the development of obesity from childhood to adulthood are important. To do so, we need to understand the factors underlying the continuity of BMI during the growth period.

Based on the previous evidence, it is likely that genetic factors are important in explaining the continuity of BMI during childhood and adolescence. Twin studies have shown that genetic factors explain a major proportion of BMI variation during childhood [10] and adulthood [11]. Environmental factors shared by co-twins affect BMI in early childhood, but their influence disappears during middle childhood and is not present at all by adolescence [10]. The importance of genetic factors on BMI is further confirmed by genome-wide-association (GWA) studies, which have identified a large number of genetic variants affecting BMI variation in childhood [12] and adulthood [13]. Previous longitudinal twin studies from different countries [14–18] have estimated genetic correlations between BMI at different ages over childhood and adolescence (r_G_ = 0.32–0.91 depending on the ages at the time of measurements). Further, a GWA meta-analysis found a strong genetic correlation of childhood BMI with adult BMI (r_G_ = 0.76) and somewhat weaker genetic correlations with waist-to-hip ratio (r_G_ = 0.39) and body-fat percentage in adulthood (r_G_ = 0.46) [12].

Even when the previous studies have reported genetic correlations between certain ages, a comprehensive set of estimates is not available because single data files do not include enough measures for all ages to estimate all these correlations with adequate power. There is evidence on the changing genetic variation from early to mid-childhood [19], which can affect genetic correlations over ages if new genetic variation emerges. The patterns of genetic correlations can give new information how genetic factors contribute to the development of BMI from infancy to adulthood. In this study, we will analyze this by using a very large twin dataset allowing us to estimate all age-to-age genetic correlations from 1 to 19 years of age separately for males and females. We also compare these genetic correlations of BMI to the genetic correlations of height. We expect that if a new genetic component affecting BMI emerges, it will modify the age pattern of BMI correlations but not height correlations. This information would be important when identifying periods in childhood most strongly associated with adulthood BMI, which can guide further interventions to prevent the development of adult obesity and subsequent health risks.

## Data and methods

The data were derived from the COllaborative project of Development of Anthropometrical measures in Twins (CODATwins) database described in detail elsewhere [20, 21]. For this study, we selected those participants having at least two longitudinal measures between 1 and 19 years of age. Together, we had 25 longitudinal twin cohorts including 82,080 twin individuals (51% females) and representing 11 countries (the cohort names are given in a footnote of Table 1). The large majority of the participants came from six European countries (Denmark, Finland, Italy, Netherlands, Sweden and the UK) representing 79% of participants. Outside Europe, the biggest representation came from Japan (9% of participants) and the USA (7% of participants). Other countries having much smaller representations were Australia (3% of participants), Canada (1% of participants), Israel (<1% of participants) and Guinea-Bissau (<1% of participants). The pooled analysis was approved by the ethical committee of Department of Public Health, University of Helsinki, and the methods were carried out in accordance with the approved guidelines. Only a limited set of observational variables and anonymized data were delivered to the data management center at University of Helsinki. All participants were volunteers and they or their parents gave informed consent when participating in their original studies. The measures were done between the years 1959–2022 and 98% of them were done after 1980. Among the participating twins, there were 38,530 complete twin pairs of which 38% were monozygotic (MZ) pairs, 33% same-sex dizygotic (SSDZ) pairs and 29% opposite-sex dizygotic (OSDZ) pairs. For these twins, we had 283,766 longitudinal height and weight measures together (the number of observations for each age and sex combination is given in Supplementary Table 1). BMI was calculated as weight in kilograms divided by the square of height in meters (kg/m^2^). The BMI distribution was normalized using log-transformation producing roughly normal distributions (skewness parameters from 0.17 to 0.85 at different ages). The height distribution was close to the normal distribution without transformation (skewness parameters from −0.14 to 0.14 at different ages). Further, we adjusted BMI and height for exact age, birth year and study cohort differences within each 1-year age and sex group by calculating regression residuals.

**Table 1:** Number of observations and means and standard deviations of body mass index (kg/m^2^) and height (cm) by age and sex^a^.

Age	Males					Females				
	*N*	BMI		Height		*N*	BMI		Height	
		Mean	SD	Mean	SD		Mean	SD	Mean	SD
1	14,860	17.1	1.40	73.9	4.43	14,978	16.8	1.42	72.5	4.47
2	12,379	16.5	1.41	86.8	4.31	12,134	16.2	1.42	85.6	4.36
3	14,835	15.9	1.42	96.1	4.40	15,279	15.7	1.48	95.1	4.50
4	7633	15.8	1.69	102.5	4.89	7737	15.7	1.81	101.3	4.86
5	6603	15.2	1.43	111.4	5.91	6564	15.1	1.54	110.7	6.05
6	2233	15.4	1.52	114.1	5.61	2065	15.3	1.60	113.2	5.57
7	11,856	15.4	1.77	124.4	6.50	12,305	15.4	1.98	123.5	6.50
8	5217	15.7	1.79	128.3	6.38	5192	15.7	2.03	127.6	6.40
9	5149	16.4	2.33	133.8	6.78	5120	16.5	2.62	133.0	6.97
10	9687	16.6	2.34	141.0	7.15	9940	16.7	2.62	140.6	7.30
11	7177	17.3	2.65	144.4	7.09	7357	17.5	2.83	144.8	7.47
12	9831	17.8	2.82	151.9	7.98	10,161	18.0	2.93	153.0	8.06
13	3474	18.4	2.90	158.4	8.94	3413	18.7	3.11	158.0	7.49
14	7250	19.5	3.07	165.7	8.84	7800	19.8	3.19	162.1	6.79
15	3270	19.9	3.20	172.1	8.42	3266	20.2	3.23	164.7	7.11
16	5540	20.7	2.89	175.7	7.39	6350	20.7	3.01	164.9	6.50
17	6125	21.2	2.86	177.8	7.12	6904	20.7	2.80	165.7	6.54
18	3692	21.7	2.98	178.8	7.28	3960	21.3	3.34	165.8	6.79
19	2901	22.0	2.87	179.3	7.29	3529	21.4	3.29	166.1	6.71

^a^Includes the following individual twin cohorts: Boston University Twin Project, Child and Adolescent Twin Study in Sweden (CATSS), Colorado Twin Registry, Danish Twin Cohort, FinnTwin12, FinnTwin16, Gemini Study, Guinea Bissau Twin Study, Japanese Twin Registry, Longitudinal Israeli Study of Twins, Michigan Twins Project, Minnesota Twin Family Study, Netherlands Twin Cohort (children), Netherlands Twin Cohort (adults), Ochanomizu University Twin Project, Peri/Postnatal Epigenetic Twins Study (PETS), Quebec Newborn Twin Study, Queensland Twin Register, Swedish Young Male Twins Study (children), Swedish Young Male Twins Study (adults), TCHAD-study, University of Southern California Twin Study, Washington State Twin Registry, Twins Early Developmental Study (TEDS), West Japan Twins and Higher Order Multiple Births Registry

The data were analyzed using classical twin modeling based on the theory of quantitative genetics utilizing the different genetic relatedness of MZ and DZ twins [22]. MZ twins have virtually the same gene sequence whereas DZ twins share, on average, half of the genetic variations in the same way as ordinary siblings. Based on this design, it is possible to decompose the trait variation into additive genetic variation (A) including the effects of all relevant loci on the trait (correlation 1 within MZ and 0.5 within DZ co-twins), shared environmental variation (C) including the effects of all environmental factors making the co-twins similar (correlation 1 within both MZ and DZ co-twins) and unique environmental variation (E) including the effects of all environmental factors making the co-twins different including measurement error (correlation 0 within both MZ and DZ co-twins). These components were estimated using OpenMx package, version 3.0.2, of R statistical software, and the 95% confidence intervals (CI) were calculated using the maximum likelihood estimation [23]. OpenMx software uses structural equation technique, and thus these components are defined as random latent factors in the model having the predefined correlation structure between co-twins. To be able to estimate these latent components, we needed to assume similar variances for MZ and DZ twins. We had previously reported that the standard deviation (SD) of BMI at some ages in childhood was somewhat higher than in MZ twins [24]. However, the difference was small, 15% or less, and become statistically significant because of the very large sample size. Thus, we needed to assume that the genetic and environmental factors explain a similar proportion of variation in MZ and DZ twins. For height, no differences in SD was found. We also found that DZ twins are slightly taller and heavier than MZ twins and thus used different means for the zygosity groups [24]. There was some evidence for sex-specific genetic effects on both BMI [10] and height [25] over childhood and adolescence, and we therefore allowed for these effects by estimating the genetic correlation in OSDZ twins, rather than constraining it at 0.5 expected for SSDZ twins.

In this study, we utilized bivariate Cholesky decomposition, which is a model free method to decompose all variation and co-variation in the data into uncorrelated latent factors [26]. This method was used to decompose the co-variation between the measures at different ages into genetic and environmental covariances. Standardizing these covariances provides us the estimates of additive genetic (r_A_), shared environmental (r_C_) and unique environmental (r_E_) correlations. The observed (phenotypic) correlation (r) between two observation (P_1_ and P_2_) at different ages is defined as r(P_1_, P_2_) = a_1_*r_A_*a_2_ + c_1_*r_C_*c_2_ + e_1_*r_E_*e_2_, where a, c and e are the square roots of the variance components A, C and E. Results for univariate models based on the same CODATwins database have been reported previously for BMI [10] and height [25]. Since we have found that shared environmental variation affected BMI variation only in early childhood [10], we applied an additive genetic/ unique environmental effect (AE) model as the main model. However, we repeated the analyses with an additive genetic/ shared environmental/unique environmental effect (ACE) model to test whether estimating the shared environmental component has an effect on the estimated genetic correlations.

## Results

Table 1 presents the means and SDs of BMI and height at each age in males and females from 1 to 19 years of age. The development of BMI and height followed the expected patterns. BMI decreased during early childhood and reached the nadir at 5 years of age both in boys and girls, and then increased until 19 years of age. Growth in height was very rapid in early childhood and again in adolescence indicating the puberty. Mean BMI was very similar in males and females at all ages. Males were taller than females at all ages except 11 and 12 years of age indicating the earlier start of puberty in females.

**Fig. 1:**
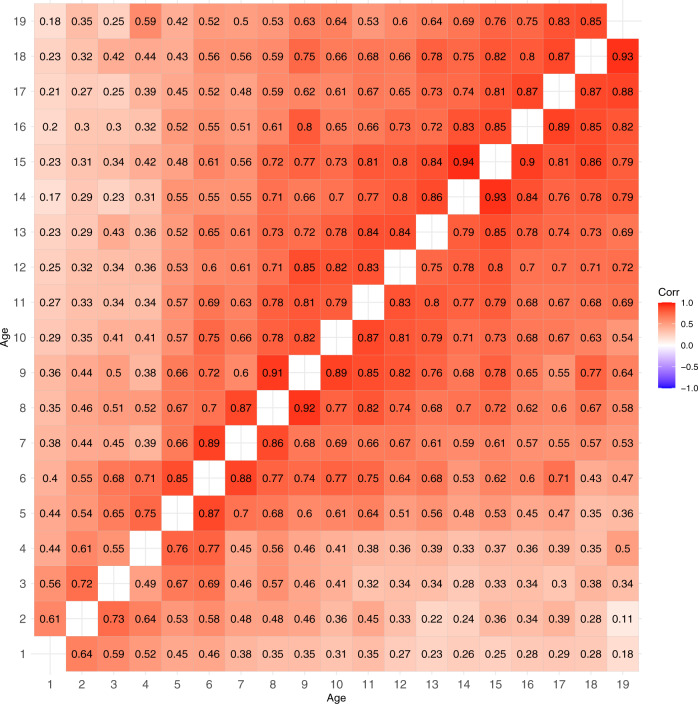
Trait correlations of BMI between different ages from 1 to 19 years in males (upper triangular matrix) and females (lower triangular matrix).

Figure 1 presents the BMI trait correlations between all age combinations in males (upper triangular matrix) and females (lower triangular matrix). The 95% CIs for these correlations are presented in Supplementary Table 2. When the age difference between the measures increased, the BMI correlations decreased. However, there was a clear change in the size of correlations after 5 years of age when the BMI correlations with later ages become systematically stronger. The size of BMI correlations was roughly similar in males and females.

**Fig. 2:**
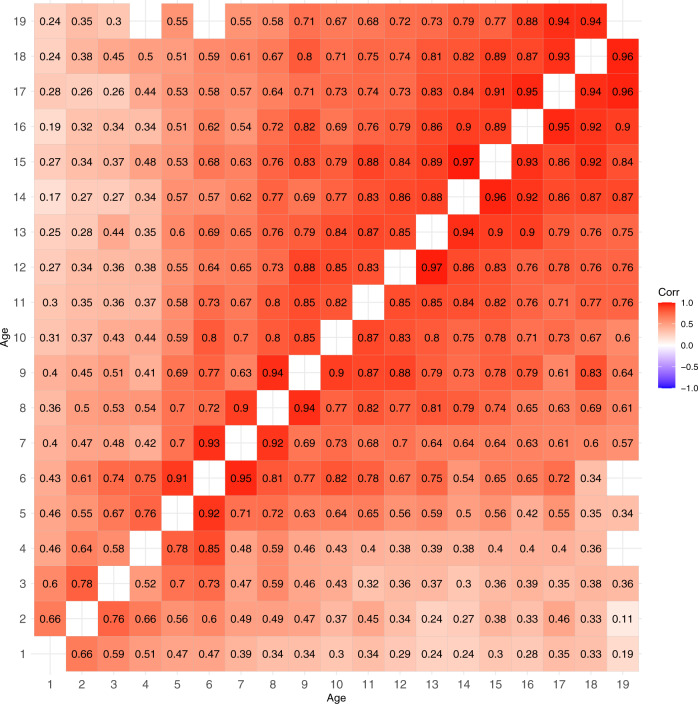
Additive genetic correlations of BMI between different ages from 1 to 19 years in males (upper triangular matrix) and females (lower triangular matrix).

**Fig. 3:**
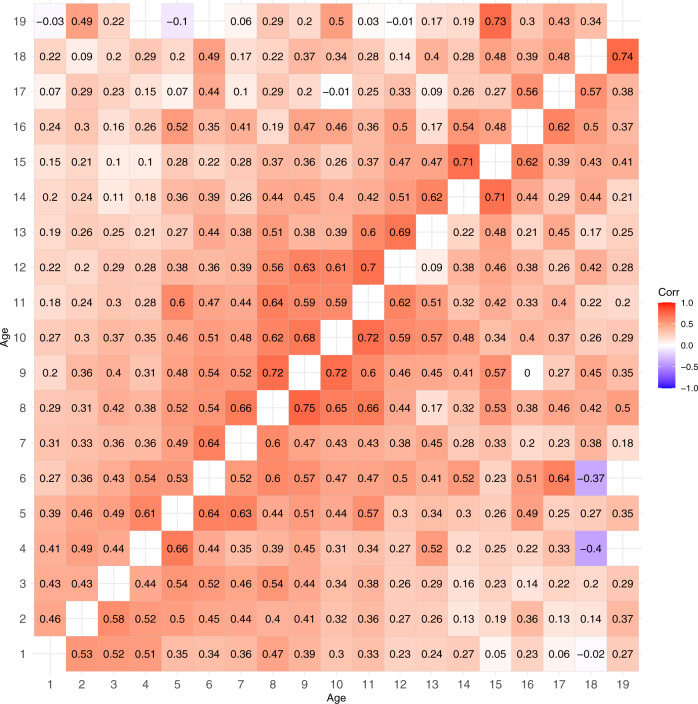
Specific environmental correlations of BMI between different ages from 1 to 19 years in males (upper triangular matrix) and females (lower triangular matrix).

Next, we decomposed these bivariate BMI trait correlations into additive genetic correlations (Fig. 2) and specific environmental correlations (Fig. 3). The 95% CIs for these correlations are given in Supplementary Tables 3 and 4, respectively. The additive genetic correlations formed a similar pattern as the trait correlations, i.e., additive genetic correlations increased after 5 years of age, but they were somewhat stronger than the trait correlations. The unique environmental correlations were weaker than additive genetic correlations but, with a few exceptions, positive.

We then conducted similar analyzes for height. Especially in infancy, the height correlations with the measures at later ages were stronger than for BMI (Supplementary Fig 1; 95% CIs presented in Supplementary Table 5) and somewhat decreased when the age between the measurements increased. Similar to BMI, the additive genetic correlations of height were somewhat stronger than the trait correlations (Supplementary Fig. 2; 95% CIs presented in Supplementary Table 6). The unique environmental correlations were somewhat weaker than additive genetic correlations but still generally higher than unique environmental correlations for BMI (Supplementary Fig. 3; 95% CIs presented in Supplementary Table 7).

Next, we tested whether the estimation of a shared environmental component, i.e. fitting the ACE model instead of the AE model, would affect the additive genetic correlations. In this model, the additive genetic correlations both for BMI (Supplementary Table 8) and height (Supplementary Table 9) were very similar to those estimated in the AE model. For BMI, the shared environmental correlations were found in early childhood, but after that they largely vanished and could not be reliably estimated: many of them were negative and 95% CIs included zero (Supplementary Table 10). For height, the shared environmental correlations were generally positive but lower than additive genetic correlations (Supplementary Table 11). Unique environmental correlations were very similar for BMI (Supplementary Table 12) and height (Supplementary Table 13) in the ACE and AE models.

Finally, we tested the universality of the patterns of genetic correlations in two geographic areas we had enough data to estimate most of the genetic correlations: Europe and Japan representing East Asia. The correlation patterns for BMI were roughly similar in Europe (Supplementary Fig. 4) and Japan (Supplementary Fig. 5) showing deceasing correlations when the age difference increased. Also for height, we did not find systematic differences between Europe (Supplementary Fig. 6) and Japan (Supplementary Fig. 7). The additive genetic correlations for height in both regions were higher than estimated for BMI.

## Discussion

In this study based on a very large, pooled twin dataset, we were able to estimate all age-to-age genetic correlations of BMI from infancy to the onset of adulthood and compare them to the corresponding correlations of height. Our results show that the continuity of BMI and height over childhood and adolescence is predominantly affected by genetic factors since the year-to-year genetic correlations were systematically higher than the corresponding trait correlations. Both for BMI and height, we found that the genetic correlations decreased when the age difference between the measures increased indicating that partly new genetic factors start to act at each age. However, this decrease was stronger for the genetic correlations of BMI than height. This may suggest that there are more changes in the genetic regulation of weight than height over childhood and adolescence. This can result from the major changes in body composition during the growth period [27], since it is known that somewhat different sets of genes affect the development of different body tissues [28]. However, these lower genetic correlations of BMI can also be affected by environmental factors if they interact with genetic factors affecting weight and thus modifying the genetic components [29].

The genetic correlations of BMI systematically increased from early to middle childhood whereas for height we did not see a clear age pattern and they were high even between infancy and adulthood. In our previous study, we found that the genetic variation of BMI increased after 5 years of age indicating that new genetic factors start to affect BMI after that age [10]. This new genetic component emerging after 5 years of age is consistent with the decreasing year-to-year genetic correlations showing partly different genetic factors affecting weight in early and middle childhood. There is also molecular level genetic evidence on changing genetic factors affecting weight from early to middle childhood. A GWA study of 300,000 participants found that the associations between the polygenic score of BMI and measures of BMI become stronger from early to middle childhood [19]. Further, the variants in the first intron of the FTO gene – the most important genetic variants associated with BMI in adulthood – are not associated with BMI in infancy and this association emerges after 4 years of age [30–32].

It is possible that the new genetic variation affecting BMI after 5 years of age is related to behavioral factors becoming more important for the individual variation of weight in middle childhood and later ages when children can more independently decide about their eating and physical activity behavior affecting the development of weight. Previous large-scale GWA studies of adult BMI have found that the expression of genetic variants associated with higher BMI are enriched in the brain – especially in the hypothalamus, pituitary gland, hippocampus and limbic system [33, 34]. These brain areas have important roles in appetite regulation, learning, cognition, emotion and memory [35]. Thus, this new genetic component can reflect the changing interaction between new environments, such as school and participation in leisure time activities, and the genetic background of the child. This is suggested, for example, by the correlations in eating behavior and physical activity between friends in childhood and adolescence [36]. For eating behavior, there is also some direct evidence for this since the polygenic risk score of BMI was found to be associated with increasing tendency of overeating over childhood measured by parental worries [37]. Also for physical activity, a common genetic component affecting at different ages was found [38]. However, there is only little direct evidence yet how physical activity is related to genetic variants associated with BMI, and the association between BMI and physical activity can also be reciprocal so that high BMI can lead to physical inactivity [39].

Interestingly, this change in the genetic correlations coincides with the timing of adiposity rebound at the age of 5 in our data. Previous studies have associated early adiposity rebound with higher risk of adult obesity [40]. A recent large GWA study found strong genetic correlations of adult BMI with the timing of adiposity rebound and BMI at that age, but much weaker correlations with timing of infancy adiposity peak at the end of the first year and BMI at that age [41]. It is thus possible that different developmental trajectories are associated with adult BMI, and these associations are contributed by genetic factors. However, we cannot study this hypothesis directly since it would have needed more detailed longitudinal measures for same individuals than available in our data.

We did not find any evidence that environmental factors shared by co-twins would have affected the continuity of BMI after early childhood. This is consistent with our previous study showing that shared environmental factors have an effect on BMI only in early childhood [10]. However, for height, shared environmental correlations were found. This finding may be because nutritional deficit, especially the lack of protein, during the two first years of life has influence on height lasting until adulthood and is thus not fully compensated if nutrition will improve later in life [42]. We have previously reported that shared environmental factors explain nearly 40% of height variation in early childhood and from 10% to 20% in middle childhood and adolescence [25]. Thus, shared environmental factors contribute to the continuity of height even when their role is weaker than for genetic factors. The lack of shared environmental effects on BMI may be surprising when considering that there is good evidence that socio-economic characteristics of childhood family affect BMI [43]. However, this result can be because the family environment may not affect BMI directly but rather interact with genetic factors; if the environmental exposure is shared by co-twins, the gene-environment interactions are modeled as additive genetic factors since MZ twins react to the environmental exposure in a more similar way than DZ twins [29]. There is previous evidence for the interaction between genetic factors and parental education based both in twin [44] and GWA studies [45]. However, in general, more research in this area is needed to estimate how much of the genetic influences involve interactions with environmental exposures.

It is also noteworthy that while specific environmental correlations were much weaker than additive genetic correlations, they were still moderate for BMI and even higher for height. As we have reported earlier, unique environmental factors explain from 10% to 20% of BMI variation in childhood and adolescence [10], and thus they do pay a role when considering factors important for BMI. These correlations demonstrate that there can be long lasting environmental exposures in childhood and adolescence affecting BMI and differing between co-twins. Identifying these factors may offer interesting opportunities to find possible targets for early life interventions. However, it is also possible that these factors reflect random variation leading to co-twins to different paths. For example, it is possible that higher BMI leads to physical inactivity that further leads to higher BMI thus reinforcing these differences [39]. Since measurement error is modeled as part of unique environmental factors in our statistical model, it could, in principle, contribute to these correlations as well. However, this would require a correlated error of measurement affecting the measures of the same twin individual done over several years.

The main limitation of our data was that we had only information on weight and height and thus could only calculate BMI. When considering heritability, BMI is conceptually problematic since it is a combination of height and several tissues contributing to weight. Thus, the genetic variation of BMI reflects the genetic variation of all these different body tissues, which are affected by partly different sets of genes [28]. This is an especially important issue for children since the body composition changes over the growth period [27]. However, BMI has found to correlate strongly with direct measures of body fat from 10 to 18 years of age [46], and a substantial amount of genetic variation is shared by BMI and waist circumference [47]. BMI can thus be regarded as a surrogate measure of direct measures of body fatness in large scale epidemiological studies. This complexity of BMI can explain why the genetic correlations of BMI in our study are lower than the genetic correlations of height. Thus, our results should be regarded as lower limits of genetic correlations of body fatness, and if using direct measures of body fat, higher estimates may be found.

Our data have also other limitations but also certain strengths. Our data did not have detailed longitudinal measures of the same individuals from infancy to adulthood. Thus, we were only able to calculate correlations but not apply, e.g., parametric growth models to analyze the growth curve patterns, which can also be associated with adult obesity [40]. Thus, our very large data do not compensate the need of repeated measures of same individuals over the growth period. Since we did not have information on adopted twins or non-twin relatives, we needed to assume random mating. There is evidence suggesting non-random mating both for BMI and height, which can lead to inflated shared environmental component if it generates a genetic correlation between spouses [48]. However, even though we found a shared environmental effect for height, we did not find it for BMI after early childhood. A limitation was also that the large majority of our datasets came from European countries and nearly all other data represented North America, Australia and East Asia. Thus, our results can be generalized only for higher-income countries and mainly for Caucasian populations. However, to study possible ethnic heterogeneity, we repeated all analyses on data from Japan representing East Asia and could not find systematic differences in the patterns of correlations as compared to European cohorts. Our main strength is the very large sample size of genetically informative data allowing us to estimate the genetic correlations for all age and sex specific combinations, i.e., 342 correlation coefficients altogether. Thus, we were able to analyze the pattern of BMI correlations from infancy to early adulthood and compare this pattern to the pattern of height correlations. This is especially important since there is no a priori hypothesis on how these correlations would vary over the growth period.

In conclusion, genetic factors are the key determinants of the continuity of BMI from childhood to the onset of adulthood. The genetic correlations decreased after middle childhood; this may indicate that new genetic factors start to affect BMI and suggests that middle childhood is an important time to prevent adult obesity. We need better understanding of the behavioral and biological pathways through which these genetic factors affect BMI thus enabling interventions for children having high genetic risk to obesity.

## Supplementary information

Supplementary information

## Data Availability

The data used in this study is owned by the third parties (the individual twin cohorts) and made available to us in condition that they will be used only in this meta-analysis. The data can be used based on the same principles as used in this study. All computer codes are freely available from the corresponding author.
