# Supplementary material for: Changing genetic architecture of body mass index from infancy to early adulthood: an individual based pooled analysis of 25 twin cohorts
Source: Int J Obes (Lond). 2022 Aug 9;46(10):1901–9. doi: 10.1038/s41366-022-01202-3 (PMC9492534; doi:10.1038/s41366-022-01202-3)
Supplement: Supplementary file 1 — Supplementary information [file 41366_2022_1202_MOESM1_ESM.pdf]

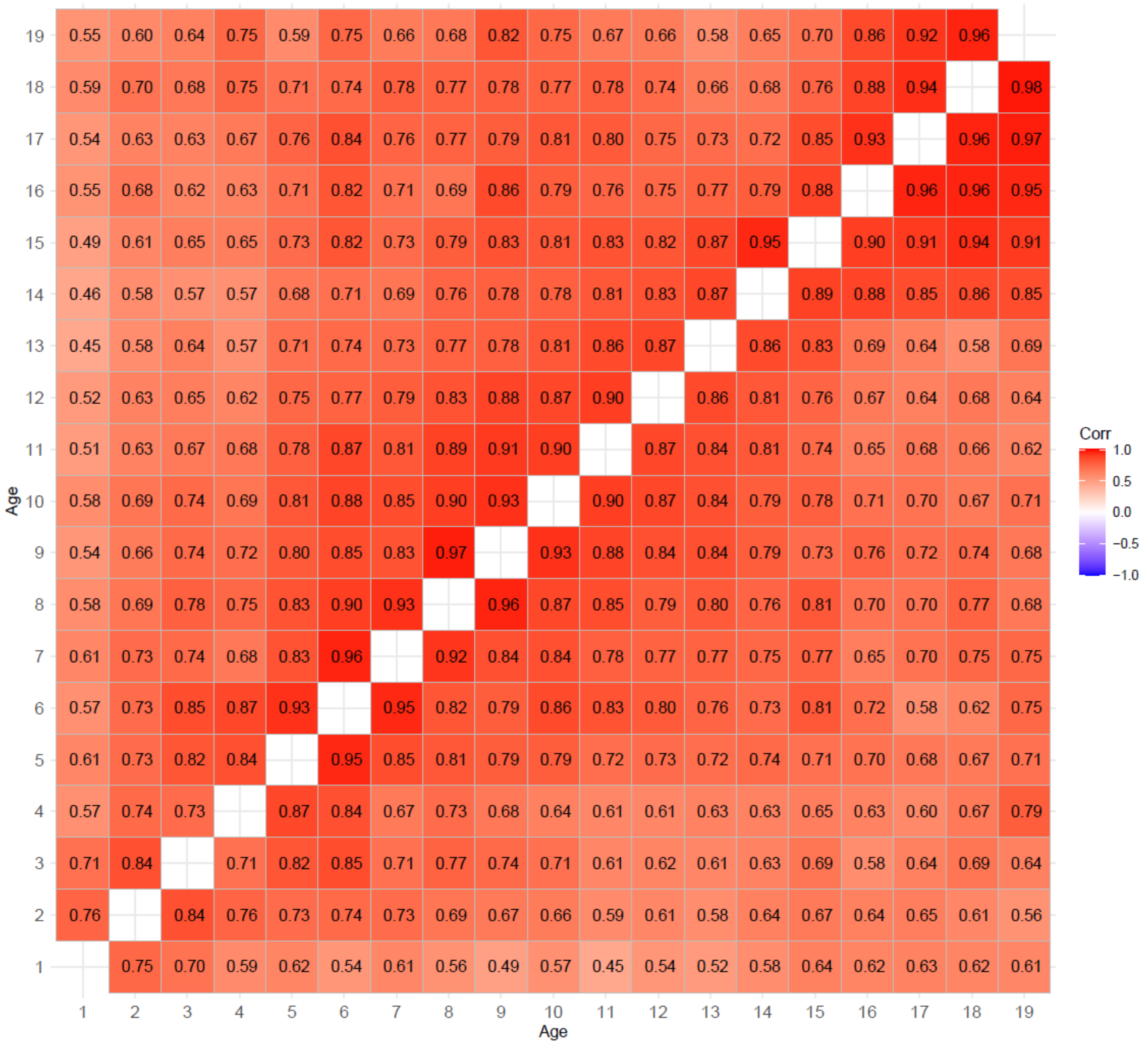

Supplementary figure 1. Trait correlations of height between different ages from 1 to 19 years of age in males (upper triangular matrix) and females (lower triangular matrix).

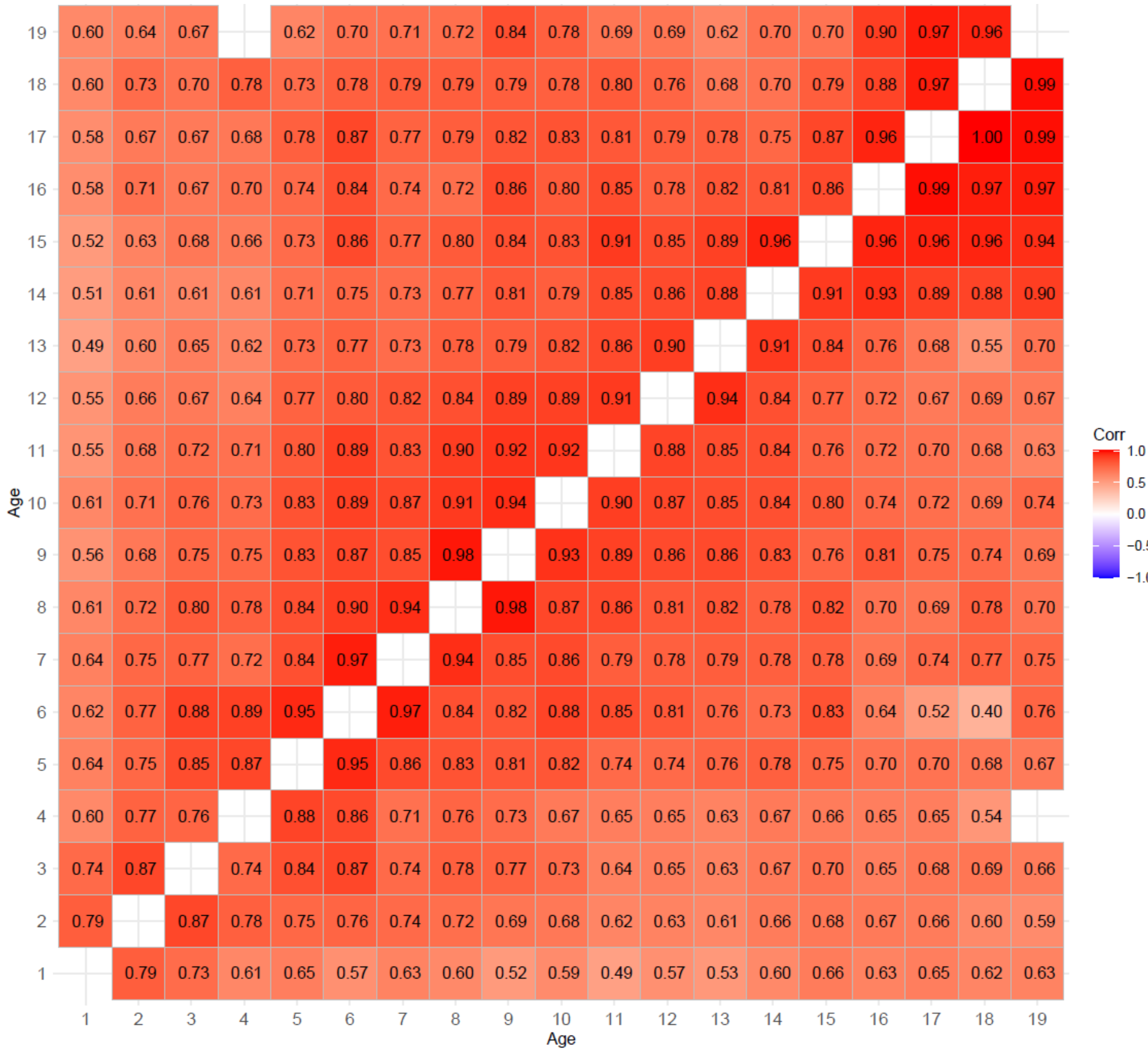

Supplementary figure 2. Additive genetic correlations of height between different ages from 1 to 19 years of age in males (upper triangular matrix) and females (lower triangular matrix).

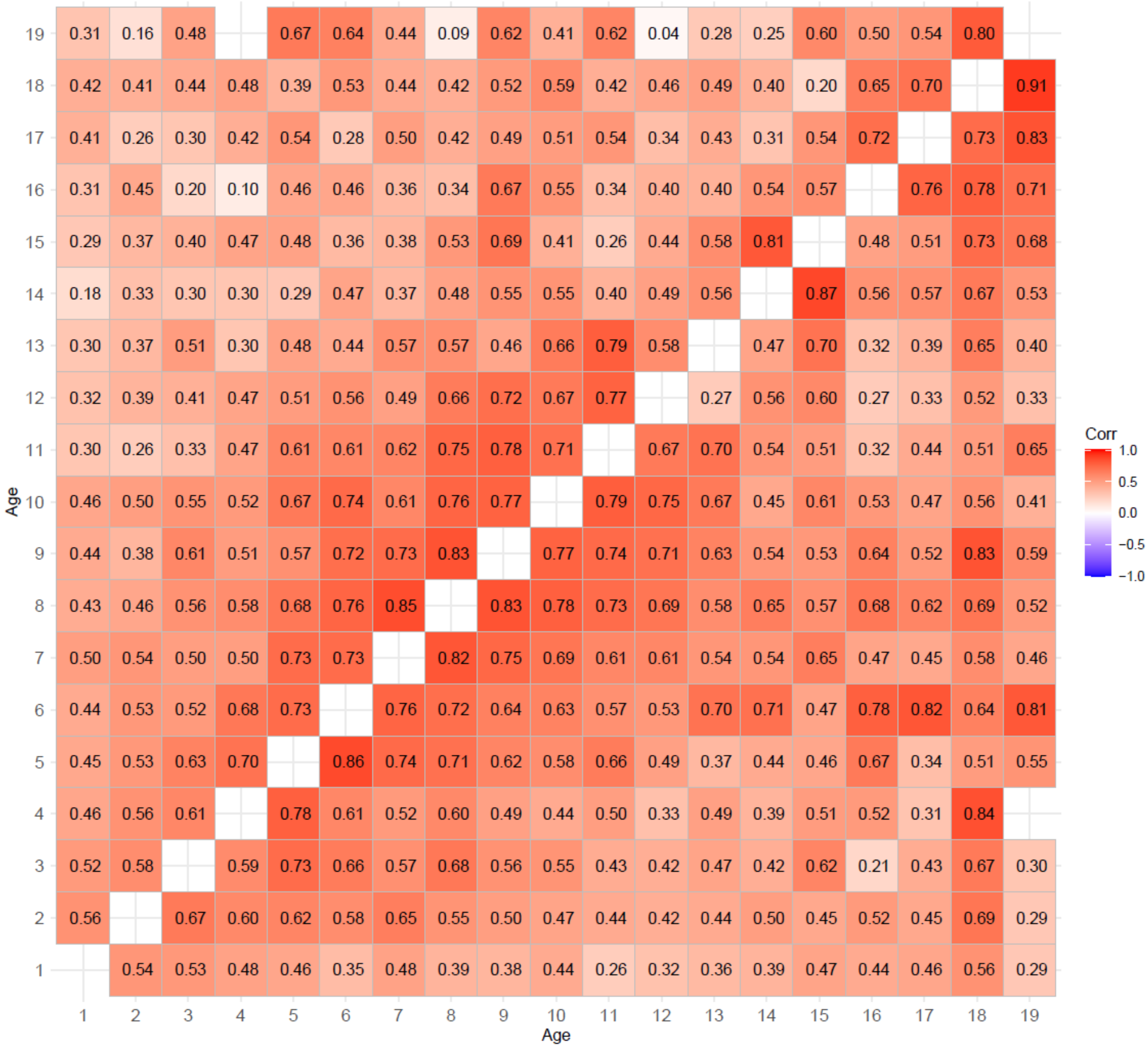

Supplementary figure 3. Unique environmental correlations of height between different ages from 1 to 19 years of age in males (upper triangular matrix) and females (lower triangular matrix).

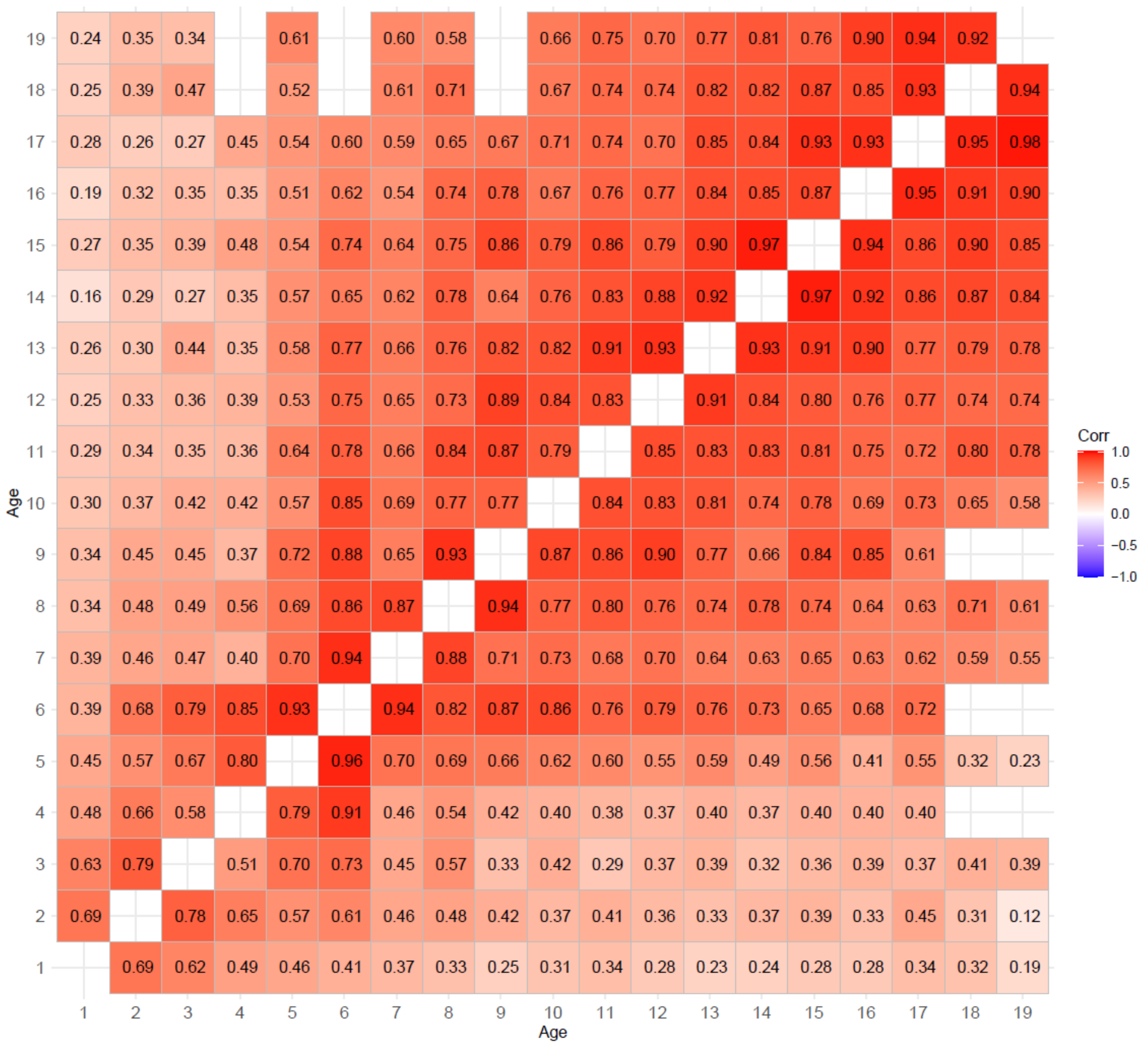

Supplementary figure 4. Additive genetic correlations of BMI between different ages from 1 to 19 years of age in males (upper triangular matrix) and females (lower triangular matrix) in European twin cohorts.

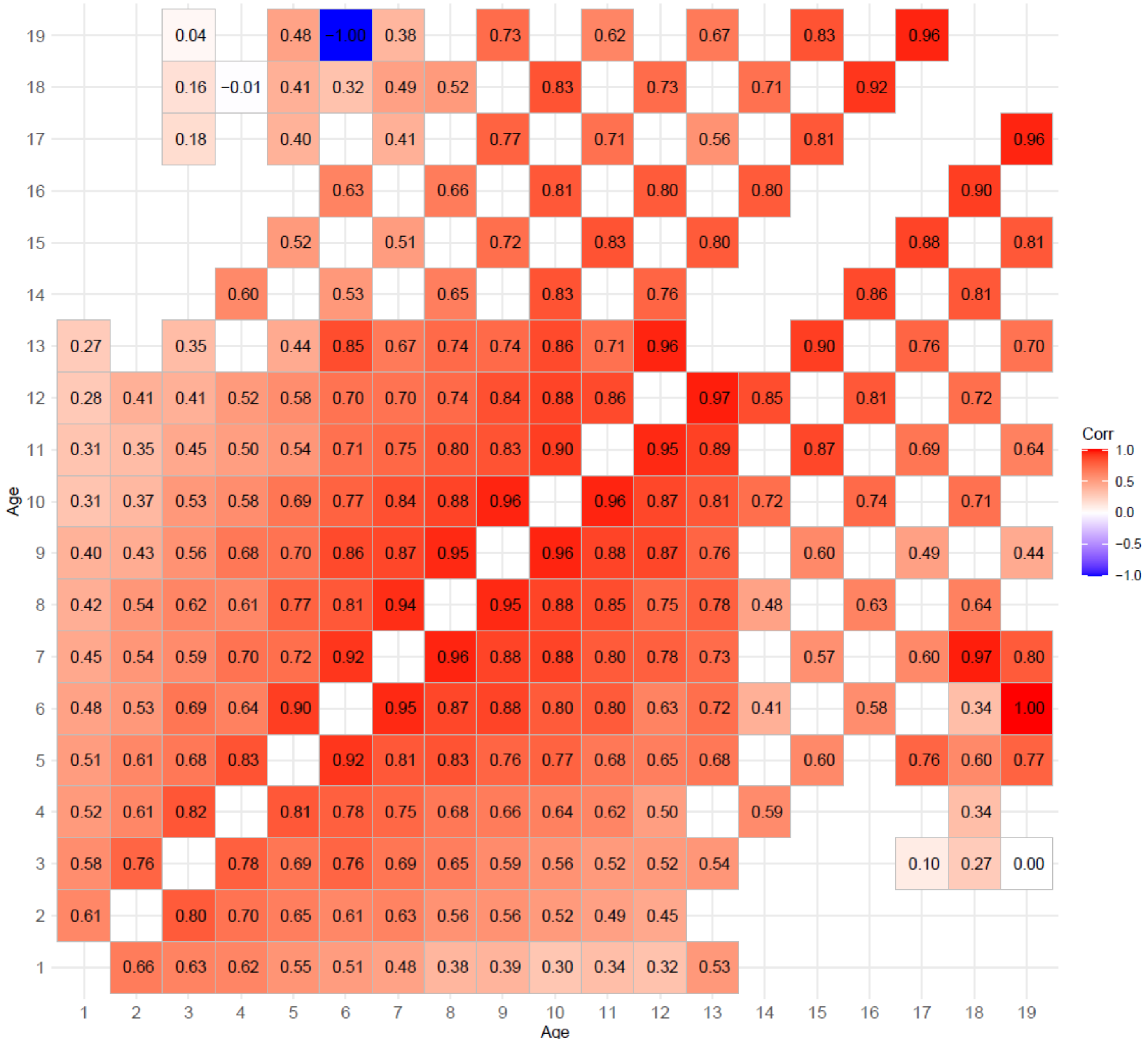

Supplementary figure 5. Additive genetic correlations of BMI between different ages from 1 to 19 years of age in males (upper triangular matrix) and females (lower triangular matrix) in Japanese twin cohorts.

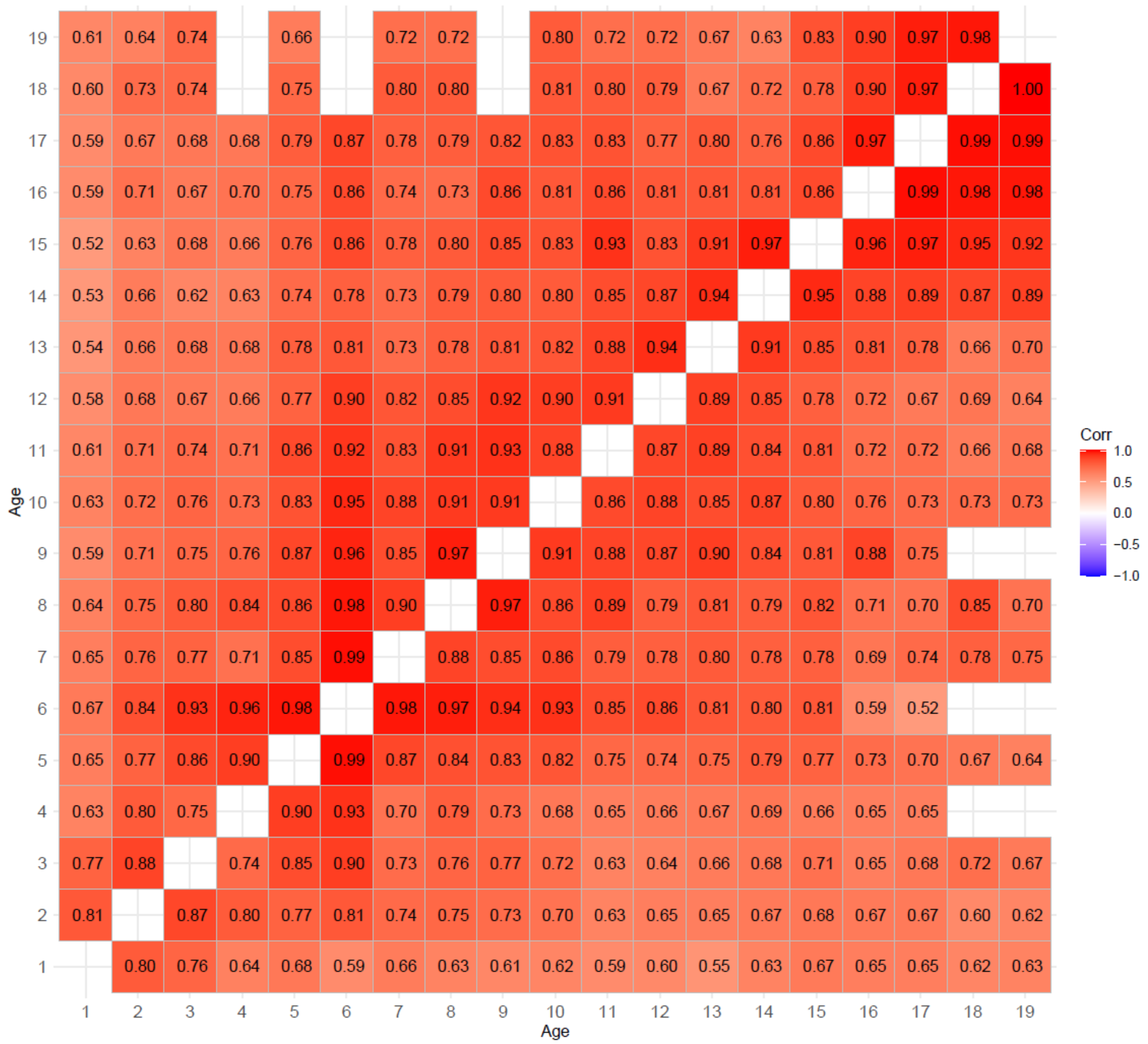

Supplementary figure 6. Additive genetic correlations of height between different ages from 1 to 19 years of age in males (upper triangular matrix) and females (lower triangular matrix) in European twin cohorts.

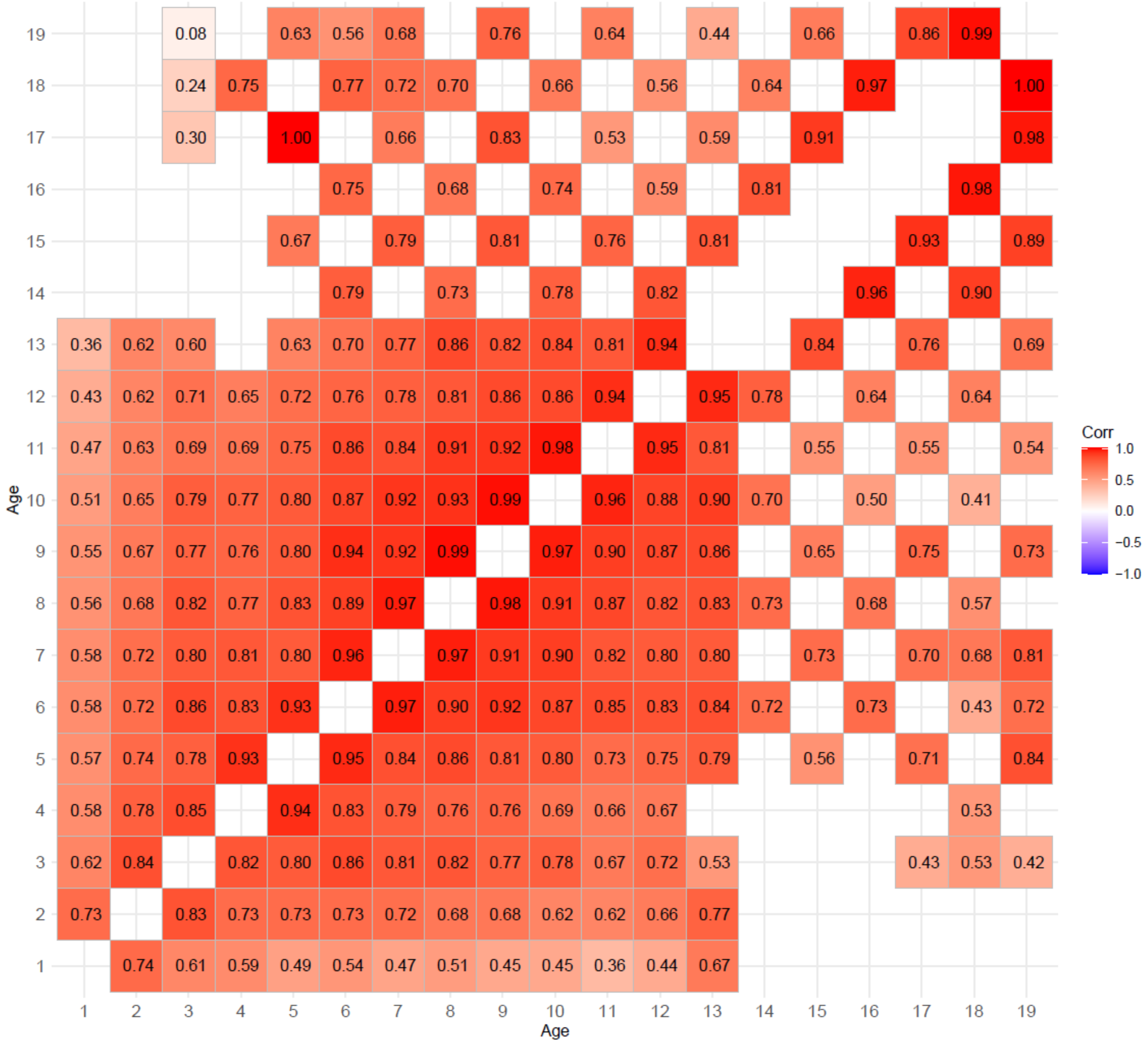

Supplementary figure 7. Additive genetic correlations of height between different ages from 1 to 19 years of age in males (upper triangular matrix) and females (lower triangular matrix) in Japanese twin cohorts.

Supplementary table 1. The number of observations for males (upper triangular matrix) and females (lower triangular matrix) for each age and sex combination from 1 to 19 years of age.

	1	2	3	4	5	6	7	8	9	10	11	12	13	14	15	16	17	18	19
19	327	190	213	42	194	36	262	232	119	276	248	378	316	297	233	1587	1439	392	
18	710	472	470	352	298	309	568	370	312	696	753	1275	423	1481	406	1122	952		364
17	947	620	1032	629	410	163	1212	371	726	890	1381	1418	539	1625	800	2453		1112	1683
16	721	527	916	715	306	243	1186	646	182	1187	608	1395	698	1367	281		2825	1270	1855
15	1372	973	1157	737	677	332	1535	605	526	1418	828	1657	845	173		231	902	392	407
14	1289	1027	1551	1258	735	401	1791	703	1031	1346	2564	2969	689		87	1407	1713	1493	327
13	1528	1173	1042	782	913	478	1476	1064	907	1217	978	625		659	693	682	507	167	396
12	4073	2600	3697	1888	2005	690	4249	1889	1105	4067	1921		561	3213	1553	1577	1502	1429	638
11	1711	1140	2860	2603	817	803	3505	1492	2061	1674		2047	830	2574	708	738	1463	601	288
10	4754	3138	4162	2452	2209	1016	5451	2452	1698		1716	4125	1095	1378	1342	1261	950	622	438
9	1670	1207	1549	1750	1029	906	2840	1301		1756	2105	1063	671	1041	358	124	654	72	135
8	2915	2112	2253	1166	1303	1194	1454		1275	2510	1444	1837	960	683	419	576	301	190	324
7	5013	3538	5525	3822	2718	996		1450	2832	5663	3673	4429	1270	1971	1445	1350	1356	332	390
6	1652	1238	1238	1367	678		858	1190	860	972	755	634	304	355	170	147	79	31	22
5	4433	3619	3545	1501		544	2786	1201	903	2301	757	2021	865	817	675	350	450	232	248
4	2436	2341	4655		1301	1173	3984	1108	1728	2556	2637	1966	646	1276	569	787	647	40	24
3	7889	7491		4611	3543	1102	5933	2223	1545	4434	3022	4007	988	1719	1149	1098	1176	332	337
2	8781		7441	2121	3523	1080	3524	2002	1159	3102	1072	2656	961	1117	839	511	626	244	274
1		8545	8085	2222	4389	1440	5105	2883	1570	4912	1677	4179	1402	1429	1342	783	995	588	549

Supplementary table 2. BMI correlations for males (upper triangular matrix) and females (lower triangular matrix) from 1 to 19 years of age.

	1	2	3	4	5	6	7	8	9	10	11	12	13	14	15	16	17	18	19
19	.18 (.08, .29)	.35 (.22, .47)	.25 (.12, .38)	.59 (.34, .75)	.42 (.30, .53)	.52 (.23, .72)	.50 (.41, .59)	.53 (.43, .62)	.67 (.55, .76)	.64 (.57, .71)	.53 (.44, .62)	.60 (.53, .66)	.64 (.57, .70)	.69 (.63, .75)	.76 (.70, .81)	.77 (.75, .79)	.83 (.82, .85)	.85 (.81, .87)	
18	.23 (.16, .30)	.32 (.23, .40)	.42 (.35, .50)	.44 (.36, .52)	.43 (.33, .52)	.56 (.48, .63)	.56 (.50, .61)	.59 (.51, .65)	.75 (.69, .79)	.66 (.61, .70)	.68 (.64, .72)	.66 (.63, .69)	.77 (.73, .81)	.75 (.72, .77)	.82 (.78, .85)	.80 (.78, .82)	.87 (.85, .88)		.93 (.92, .95)
17	.21 (.15, .27)	.27 (.20, .34)	.25 (.19, .31)	.40 (.33, .46)	.45 (.36, .52)	.52 (.40, .63)	.49 (.44, .53)	.59 (.52, .65)	.63 (.58, .67)	.61 (.57, .65)	.67 (.64, .70)	.65 (.62, .68)	.73 (.68, .76)	.76 (.74, .78)	.81 (.78, .83)	.87 (.86, .88)		.87 (.85, .88)	.88 (.87, .89)
16	.19 (.12, .26)	.30 (.22, .37)	.31 (.25, .37)	.32 (.25, .38)	.52 (.44, .60)	.55 (.46, .63)	.51 (.47, .56)	.62 (.57, .67)	.80 (.74, .84)	.65 (.61, .68)	.66 (.61, .70)	.73 (.71, .76)	.74 (.70, .77)	.83 (.81, .85)	.85 (.81, .88)		.89 (.88, .90)	.85 (.83, .86)	.82 (.81, .84)
15	.23 (.18, .28)	.31 (.25, .36)	.35 (.30, .40)	.42 (.36, .48)	.48 (.42, .53)	.61 (.54, .67)	.56 (.52, .59)	.72 (.67, .75)	.77 (.73, .80)	.73 (.70, .75)	.81 (.79, .83)	.80 (.78, .82)	.84 (.82, .86)	.94 (.92, .95)		.90 (.87, .92)	.81 (.78, .83)	.86 (.83, .89)	.79 (.75, .82)
14	.17 (.12, .22)	.29 (.24, .35)	.24 (.19, .29)	.31 (.26, .36)	.55 (.50, .60)	.55 (.48, .62)	.56 (.53, .59)	.71 (.68, .75)	.68 (.64, .71)	.70 (.68, .73)	.77 (.76, .79)	.80 (.79, .82)	.86 (.84, .88)		.93 (.89, .95)	.84 (.83, .86)	.78 (.76, .79)	.78 (.76, .80)	.79 (.74, .82)
13	.23 (.19, .28)	.29 (.24, .34)	.43 (.38, .48)	.36 (.30, .42)	.52 (.47, .57)	.65 (.60, .70)	.62 (.58, .65)	.73 (.70, .76)	.73 (.70, .76)	.79 (.77, .81)	.84 (.82, .86)	.85 (.82, .87)		.79 (.76, .82)	.85 (.83, .87)	.78 (.75, .81)	.74 (.70, .78)	.73 (.65, .79)	.69 (.64, .74)
12	.25 (.22, .28)	.32 (.28, .35)	.35 (.32, .38)	.36 (.32, .40)	.53 (.49, .56)	.60 (.55, .64)	.61 (.59, .62)	.71 (.69, .73)	.85 (.83, .86)	.82 (.81, .83)	.83 (.81, .84)		.75 (.72, .79)	.78 (.77, .80)	.80 (.78, .82)	.70 (.68, .73)	.70 (.67, .72)	.71 (.69, .74)	.72 (.68, .75)
11	.27 (.23, .32)	.33 (.28, .38)	.34 (.31, .37)	.35 (.31, .38)	.57 (.52, .62)	.69 (.65, .72)	.63 (.61, .65)	.78 (.76, .80)	.81 (.80, .83)	.79 (.78, .81)		.84 (.82, .85)	.80 (.77, .82)	.77 (.75, .78)	.79 (.76, .81)	.68 (.63, .71)	.67 (.64, .70)	.68 (.63, .72)	.69 (.62, .74)
10	.29 (.27, .32)	.35 (.32, .38)	.41 (.39, .44)	.41 (.38, .45)	.57 (.54, .59)	.75 (.73, .78)	.67 (.65, .68)	.79 (.77, .80)	.82 (.81, .84)		.87 (.86, .88)	.81 (.79, .82)	.79 (.77, .81)	.71 (.68, .73)	.73 (.71, .76)	.68 (.64, .70)	.67 (.63, .70)	.63 (.58, .67)	.54 (.47, .61)

9	.36 (.32, .40)	.44 (.39, .48)	.50 (.46, .53)	.38 (.34, .42)	.66 (.62, .69)	.72 (.69, .75)	.63 (.61, .65)	.91 (.90, .92)		.89 (.88, .90)	.85 (.83, .86)	.82 (.80, .84)	.76 (.72, .79)	.69 (.66, .72)	.78 (.73, .82)	.65 (.54, .74)	.57 (.51, .62)	.77 (.65, .85)	.64 (.53, .73)
8	.35 (.31, .38)	.46 (.43, .50)	.51 (.48, .54)	.52 (.48, .56)	.67 (.64, .70)	.70 (.67, .73)	.87 (.85, .88)		.92 (.91, .93)	.79 (.78, .81)	.82 (.80, .83)	.74 (.72, .76)	.68 (.64, .71)	.70 (.65, .73)	.72 (.67, .77)	.61 (.56, .66)	.60 (.53, .67)	.67 (.58, .74)	.58 (.50, .65)
7	.38 (.35, .40)	.44 (.42, .47)	.46 (.44, .48)	.39 (.36, .42)	.66 (.64, .68)	.89 (.87, .90)		.86 (.85, .88)	.71 (.69, .73)	.70 (.68, .71)	.67 (.66, .69)	.67 (.66, .69)	.62 (.58, .65)	.59 (.56, .62)	.61 (.58, .64)	.57 (.53, .60)	.55 (.51, .58)	.57 (.49, .64)	.53 (.45, .60)
6	.40 (.35, .44)	.55 (.51, .59)	.68 (.65, .71)	.71 (.69, .74)	.85 (.83, .87)		.88 (.86, .89)	.77 (.75, .80)	.74 (.71, .77)	.77 (.74, .79)	.75 (.71, .78)	.64 (.59, .68)	.68 (.61, .74)	.53 (.45, .60)	.62 (.52, .70)	.60 (.49, .70)	.71 (.58, .81)	.43 (.09, .68)	.47 (.06, .75)
5	.45 (.42, .47)	.54 (.51, .56)	.64 (.63, .66)	.75 (.73, .77)		.87 (.85, .89)	.70 (.68, .72)	.69 (.66, .72)	.60 (.56, .64)	.61 (.58, .63)	.64 (.60, .68)	.51 (.48, .55)	.56 (.51, .60)	.48 (.42, .53)	.53 (.48, .58)	.45 (.36, .53)	.47 (.40, .54)	.35 (.23, .46)	.36 (.25, .46)
4	.47 (.44, .50)	.61 (.59, .64)	.56 (.54, .58)		.76 (.73, .78)	.77 (.75, .79)	.46 (.43, .48)	.56 (.52, .60)	.46 (.42, .49)	.41 (.38, .44)	.38 (.35, .41)	.37 (.33, .40)	.39 (.32, .45)	.34 (.30, .39)	.37 (.30, .44)	.36 (.30, .42)	.39 (.32, .45)	.35 (.04, .60)	.50 (.12, .75)
3	.58 (.56, .59)	.73 (.72, .74)		.49 (.47, .52)	.67 (.65, .69)	.69 (.66, .72)	.46 (.44, .48)	.57 (.55, .60)	.46 (.42, .50)	.42 (.39, .44)	.32 (.29, .36)	.35 (.32, .38)	.34 (.28, .39)	.30 (.26, .34)	.33 (.27, .38)	.34 (.29, .39)	.31 (.26, .36)	.38 (.28, .47)	.34 (.24, .43)
2	.62 (.61, .64)		.75 (.74, .76)	.64 (.61, .66)	.53 (.51, .55)	.58 (.54, .62)	.48 (.45, .50)	.48 (.44, .51)	.46 (.41, .50)	.36 (.33, .39)	.45 (.40, .50)	.33 (.30, .37)	.22 (.16, .28)	.24 (.18, .29)	.36 (.30, .42)	.34 (.26, .42)	.39 (.32, .46)	.28 (.16, .40)	.11 (.00, .23)
1		.65 (.63, .66)	.61 (.59, .62)	.52 (.49, .55)	.45 (.42, .47)	.46 (.41, .50)	.38 (.36, .41)	.35 (.32, .38)	.35 (.30, .39)	.31 (.28, .33)	.35 (.30, .39)	.28 (.25, .30)	.23 (.18, .28)	.26 (.21, .30)	.25 (.20, .30)	.28 (.22, .35)	.30 (.24, .36)	.28 (.20, .35)	.18 (.10, .26)

Supplementary table 3. Additive genetic correlations of BMI for males (upper triangular matrix) and females (lower triangular matrix) from 1 to 19 years of age.

	1	2	3	4	5	6	7	8	9	10	11	12	13	14	15	16	17	18	19
19	.24 (.08, .38)	.35 (.16, .50)	.30 (.12, .46)	NA (NA, NA)	.55 (.39, .68)	NA (NA, NA)	.55 (.42, .67)	.58 (.45, .69)	.74 (.60, .85)	.67 (.57, .75)	.68 (.56, .79)	.72 (.63, .80)	.73 (.63, .82)	.79 (.71, .86)	.77 (.68, .83)	.90 (.87, .92)	.94 (.91, .96)	.94 (.90, .97)	
18	.24 (.14, .34)	.38 (.26, .49)	.45 (.35, .54)	.50 (.38, .61)	.51 (.37, .63)	.59 (NA, NA)	.61 (.52, .68)	.67 (.59, .75)	.80 (.74, .86)	.71 (.65, .76)	.75 (.69, .79)	.74 (.70, .78)	.81 (.75, .86)	.82 (.79, .85)	.89 (.84, .93)	.87 (.84, .90)	.93 (.91, .96)		.96 (.95, .98)
17	.28 (.19, .36)	.26 (.15, .37)	.26 (.18, .34)	.45 (.36, .54)	.53 (.42, .64)	.58 (.54, .69)	.58 (.52, .64)	.64 (.55, .72)	.72 (.66, .78)	.73 (.67, .78)	.74 (.70, .78)	.73 (.69, .77)	.83 (.78, .87)	.86 (.83, .89)	.91 (.88, .94)	.95 (.93, .96)		.94 (.91, .96)	.96 (.95, .98)
16	.19 (.09, .29)	.32 (.20, .42)	.35 (.26, .44)	.34 (.24, .44)	.51 (.38, .62)	.62 (.49, .72)	.54 (.48, .60)	.73 (.67, .79)	.83 (.74, .89)	.69 (.64, .73)	.76 (.68, .82)	.79 (.75, .82)	.88 (.83, .92)	.90 (.88, .92)	.89 (.83, .94)		.95 (.94, .96)	.92 (.90, .94)	.90 (.88, .92)
15	.27 (.20, .34)	.34 (.26, .42)	.39 (.32, .45)	.48 (.40, .55)	.53 (.45, .61)	.68 (.58, .76)	.63 (.59, .68)	.76 (.70, .80)	.83 (.78, .86)	.79 (.76, .82)	.88 (.85, .90)	.84 (.82, .86)	.89 (.86, .91)	.97 (.94, .98)		.93 (.88, .96)	.87 (.83, .90)	.92 (.89, .95)	.84 (.80, .88)
14	.17 (.09, .24)	.26 (.19, .34)	.28 (.22, .35)	.34 (.27, .40)	.57 (.50, .63)	.57 (.48, .66)	.62 (.58, .66)	.77 (.72, .81)	.70 (.65, .74)	.77 (.73, .80)	.84 (.82, .86)	.86 (.85, .88)	.88 (.85, .90)		.96 (.92, .99)	.92 (.90, .94)	.86 (.83, .88)	.87 (.84, .90)	.87 (.81, .92)
13	.25 (.19, .31)	.28 (.21, .35)	.44 (.38, .51)	.34 (.25, .42)	.59 (.53, .66)	.69 (.61, .75)	.65 (.61, .69)	.76 (.72, .79)	.79 (.75, .83)	.83 (.80, .85)	.87 (.84, .89)	0 .86 (NA, .89)		.94 (.91, .97)	.90 (.87, .92)	.90 (.86, .93)	.79 (.73, .84)	.76 (.64, .86)	.75 (.68, .81)
12	.26 (.22, .30)	.34 (.30, .38)	.37 (.33, .41)	.39 (.33, .44)	.56 (.51, .59)	.64 (.57, .70)	.65 (.63, .67)	.73 (.70, .76)	.88 (.86, .90)	.85 (.84, .86)	.83 (.81, .85)		.97 (.93, NA)	.86 (.84, .88)	.83 (.81, .85)	.76 (.73, .79)	.78 (.75, .82)	.76 (.72, .79)	.76 (.71, .81)
11	.31 (.25, .36)	.35 (.28, .41)	.36 (.32, .40)	.37 (.33, .41)	.58 (.52, .64)	.73 (.68, .78)	.68 (.65, .70)	.80 (.78, .83)	.86 (.84, .87)	.82 (.79, .84)		.87 (.85, .88)	.85 (.81, .87)	.84 (.82, .86)	.82 (.79, .86)	.76 (.70, .80)	.71 (.67, .75)	.77 (.71, .82)	.76 (.68, .83)
10	.31 (.27, .34)	.37 (.33, .41)	.43 (.40, .46)	.44 (.40, .48)	.59 (.56, .63)	.80 (.76, .83)	.71 (.69, .72)	.81 (.79, .83)	.85 (.83, .87)		.87 (.86, .89)	.83 (.82, .85)	.80 (.78, .83)	.74 (.71, .77)	.78 (.75, .81)	.71 (.66, .74)	.73 (.68, .77)	.67 (.61, .73)	.60 (.50, .69)

9	.40 (.34, .45)	.45 (.39, .50)	.51 (.46, .56)	.41 (.36, .46)	.69 (.65, .73)	.77 (.73, .80)	.65 (.62, .68)	.94 (.93, .95)		.91 (.90, .92)	.87 (.86, .89)	.88 (.86, .91)	.79 (.75, .83)	.73 (.69, .77)	.78 (.72, .83)	.79 (.65, .90)	.60 (.53, .67)	.83 (.66, .96)	.64 (.50, .75)
8	.36 (.32, .40)	.50 (.46, .54)	.53 (.49, .57)	.54 (.48, .59)	.70 (.66, .74)	.72 (.69, .76)	.90 (.88, .92)		.94 (.93, .95)	.81 (.79, .83)	.82 (.80, .85)	.77 (.75, .80)	.81 (.77, .85)	.79 (.73, .83)	.74 (.68, .79)	.65 (.58, .71)	.63 (.53, .72)	.69 (.57, .78)	.61 (.51, .70)
7	.40 (.37, .43)	.47 (.43, .50)	.49 (.46, .51)	.42 (.38, .45)	.70 (.67, .72)	.93 (.91, .94)		.92 (.90, .93)	.73 (.71, .76)	.74 (.72, .76)	.70 (.68, .72)	.71 (.69, .73)	.65 (.61, .69)	.63 (.59, .67)	.64 (.60, .68)	.63 (.58, .68)	.61 (.56, .66)	.60 (.50, .69)	.57 (.47, .66)
6	.43 (.37, .48)	.61 (.56, .66)	.74 (.70, .77)	.75 (.71, .78)	.91 (.88, .93)		.95 (.93, .96)	.81 (.78, .84)	.77 (.73, .81)	.82 (.79, .85)	.78 (.74, .82)	.67 (.60, .72)	.75 (.67, .82)	.54 (.42, .64)	.65 (.52, .76)	.65 (.65, .78)	.72 (.70, .85)	.34 (NA, NA)	NA (NA, NA)
5	.46 (.43, .49)	.55 (.52, .58)	.67 (.65, .70)	.76 (.73, .79)		.92 (.90, .95)	.71 (.69, .74)	.73 (.70, .77)	.63 (.57, .68)	.64 (.61, .67)	.65 (.59, .70)	.56 (.51, .60)	.59 (.53, .65)	.50 (.42, .57)	.56 (.48, .63)	.42 (.30, .53)	.56 (.46, .64)	.35 (.18, .49)	.34 (.19, .48)
4	.49 (.44, .53)	.64 (.61, .67)	.59 (.56, .61)		.78 (.75, .81)	.85 (.82, .88)	.48 (.45, .51)	.59 (.53, .64)	.46 (.41, .50)	.43 (.38, .47)	.40 (.36, .44)	.39 (.35, .44)	.39 (.30, .47)	.38 (.31, .44)	.40 (.31, .48)	.40 (.32, .48)	.40 (.31, .48)	.36 (-.04, .68)	NA (NA, NA)
3	.62 (.60, .63)	.78 (.77, .80)		.53 (.50, .55)	.70 (.68, .72)	.73 (.69, .77)	.48 (.45, .50)	.59 (.55, .62)	.46 (.41, .52)	.43 (.40, .46)	.33 (.29, .37)	.38 (.34, .41)	.37 (.30, .44)	.31 (.26, .37)	.36 (.29, .42)	.39 (.31, .46)	.35 (.28, .42)	.38 (.24, .50)	.36 (.23, .48)
2	.67 (.65, .68)		.78 (.77, .79)	.66 (.62, .69)	.55 (.52, .58)	.60 (.55, .65)	.49 (.45, .52)	.49 (.45, .53)	.47 (.41, .53)	.37 (.33, .41)	.45 (.38, .51)	.34 (.30, .39)	.24 (.16, .31)	.27 (.20, .35)	.38 (.30, .45)	.33 (.22, .44)	.46 (.36, .54)	.33 (.17, .48)	.11 (-.07, .28)
1		.67 (.65, .68)	.62 (.60, .64)	.51 (.46, .55)	.47 (.44, .50)	.47 (.41, .53)	.39 (.36, .42)	.34 (.29, .38)	.34 (.28, .40)	.31 (.27, .34)	.34 (.28, .40)	.29 (.25, .33)	.25 (.18, .31)	.25 (.18, .31)	.30 (.23, .36)	.28 (.18, .37)	.33 (.25, .41)	.33 (.22, .43)	.19 (.08, .30)

Supplementary table 4. Specific environmental correlations of BMI for males (upper triangular matrix) and females (lower triangular matrix) from 1 to 19 years of age.

	1	2	3	4	5	6	7	8	9	10	11	12	13	14	15	16	17	18	19
19	-.03 (-.25, .19)	.49 (.19, .71)	.22 (-.06, .48)	NA (NA, NA)	-.10 (-.37, .19)	NA (NA, NA)	.06 (-.20, .31)	.29 (-.00, .53)	.24 (-.10, .53)	.50 (.28, .67)	.03 (-.21, .28)	-.01 (-.22, .21)	.17 (-.08, .39)	.19 (-.04, .41)	.73 (.58, .83)	.33 (.22, .42)	.43 (.33, .53)	.34 (.17, .50)	
18	.20 (.06, .34)	.09 (-.08, .25)	.20 (.03, .37)	.29 (.11, .46)	.20 (-.02, .41)	.49 (NA, NA)	.17 (.01, .32)	.22 (.02, .40)	.37 (.17, .54)	.34 (.20, .46)	.28 (.12, .42)	.14 (.02, .25)	.40 (.23, .54)	.28 (.17, .39)	.48 (.31, .62)	.39 (.27, .49)	.48 (.35, .59)		.74 (.64, .81)
17	.07 (-.07, .21)	.29 (.13, .44)	.23 (.10, .36)	.15 (-.04, .34)	.07 (-.14, .28)	.44 (.14, .67)	.11 (-.01, .23)	.29 .08, .48)	.21 (.07, .35)	-.01 (-.15, .13)	.25 (.13, .36)	.34 (.23, .43)	.09 (-.07, .25)	.28 (.17, .38)	.27 (.14, .40)	.56 (.49, .62)		.57 (.48, .65)	.38 (.30, .47)
16	.22 (.08, .36)	.30 (.14, .45)	.17 (.03, .30)	.26 (.12, .40)	.52 (.32, .68)	.35 (.13, .53)	.41 (.31, .50)	.21 (.06, .36)	.47 (.20, .67)	.46 (.36, .54)	.36 (.22, .50)	.50 (.41, .57)	.21 (.07, .34)	.54 (.46, .62)	.48 (.26, .65)		.62 (.56, .66)	.50 (.40, .59)	.37 (.28, .46)
15	.15 (.03, .26)	.21 (.08, .34)	.10 (-.03, .23)	.10 (-.05, .24)	.28 (.12, .43)	.22 (.02, .41)	.28 (.18, .38)	.37 (.22, .51)	.36 (.22, .49)	.26 (.15, .36)	.37 (.25, .48)	.47 (.39, .55)	.47 (.36, .57)	.71 (.55, .82)		.62 (.40, .77)	.34 (.22, .46)	.43 (.28, .57)	.41 (.24, .55)
14	.19 (.07, .30)	.26 (.13, .38)	.11 (.00, .22)	.18 (.06, .30)	.36 (.22, .49)	.39 (.20, .55)	.27 (.18, .36)	.44 (.31, .56)	.50 (.39, .59)	.40 (.31, .49)	.42 (.35, .49)	.53 (.47, .58)	.62 (.50, .71)		.71 (.44, .86)	.44 (.34, .52)	.35 (.26, .43)	.44 (.36, .52)	.21 (-.01, .41)
13	.19 (.08, .29)	.26 (.14, .37)	.25 (.12, .37)	.27 (.11, .41)	.26 (.13, .39)	.44 (.27, .58)	.38 (.28, .47)	.51 (.41, .60)	.40 (.28, .51)	.44 (.34, .54)	.60 (.51, .68)	.70 (NA, .78)		.22 (.09, .35)	.48 (.36, .59)	.21 (.06, .34)	.45 (.32, .57)	.17 (-.12, .43)	.25 (.05, .42)
12	.23 (.17, .29)	.20 (.12, .28)	.29 (.22, .35)	.28 (.19, .37)	.38 (.30, .46)	.36 (.23, .48)	.39 (.34, .45)	.56 (.50, .62)	.63 (.56, .69)	.61 (.57, .65)	.69 (.64, .74)		.09 (NA, .23)	.39 (.32, .45)	.46 (.37, .53)	.38 (.28, .46)	.27 (.17, .36)	.42 (.32, .51)	.28 (.13, .42)
11	.18 (.09, .27)	.24 (.13, .34)	.29 (.22, .36)	.28 (.20, .36)	.60 (.50, .68)	.47 (.36, .56)	.44 (.38, .49)	.64 (.58, .69)	.58 (.53, .64)	.59 (.52, .65)		.62 (.56, .67)	.51 (.41, .59)	.34 (.27, .41)	.42 (.28, .54)	.33 (.20, .45)	.40 (.30, .49)	.22 (.05, .37)	.20 (-.04, .42)
10	.26 (.20, .32)	.30 (.23, .37)	.37 (.31, .43)	.35 (.27, .42)	.46 (.39, .53)	.51 (.42, .59)	.48(.43, .52)	.63 (.58, .68)	.68 (.63, .72)		.72 (.67, .76)	.59 (.55, .63)	.57 .48, .65)	.52 (.44, .60)	.34 (.23, .44)	.40 (.30, .50)	.37 (.25, .48)	.26 (.10, .41)	.29 (.11, .45)

9	.20 (.11, .29)	.36 (.26, .45)	.40 (.32, .48)	.31 (.22, .40)	.48 (.39, .57)	.54 (.45, .62)	.52 (.46, .57)	.72 (.67, .76)		.73 (.68, .77)	.60 (.55, .65)	.46 (.37, .54)	.45 (.33, .57)	.41 (.30, .51)	.57 (.41, .69)	.00 (-.31, .32)	.30 (.16, .44)	.45 (.03, .74)	.35 (.01, .62)
8	.29 (.22, .35)	.31 (.22, .38)	.42 (.35, .49)	.38 (.28, .48)	.52 (.43, .59)	.54 (.46, .61)	.66 (.60, .71)		.75 (.71, .79)	.66 (.61, .70)	.66 (.60, .71)	.44 (.37, .51)	.17 (.06, .29)	.32 (.18, .45)	.53 (.36, .66)	.38 (.22, .52)	.46 (.25, .62)	.42 (.16, .62)	.50 (.32, .65)
7	.31 (.26, .37)	.33 (.27, .40)	.37 (.32, .42)	.36 (.30, .42)	.49 (.43, .55)	.64 (.57, .70)		.60 (.54, .66)	.47 (.41, .52)	.43 (.39, .47)	.43 (.37, .48)	.38 (.33, .44)	.45 (.35, .54)	.31 (.22, .39)	.33 (.23, .43)	.20 (.09, .30)	.21 (.10, .32)	.38 (.17, .56)	.18 (-.03, .37)
6	.27 (.18, .35)	.36 (.26, .45)	.43 (.34, .51)	.54 (.47, .61)	.53 (.43, .62)		.52 (.43, .60)	.60 (.53, .66)	.57 (.49, .65)	.47 (.38, .56)	.47 (.36, .56)	.50 (.38, .60)	.41 (.20, .58)	.52 (.33, .67)	.23 (-.09, .51)	.51 (.23, .71)	.64 (.23, .86)	-.37 (NA, NA)	NA (NA, NA)
5	.39 (.33, .44)	.46 (.40, .51)	.49 (.43, .54)	.61 (.54, .67)		.64 (.55, .72)	.63 (.58, .67)	.43 (.34, .52)	.51 (.41, .60)	.44 (.37, .51)	.57 (.48, .65)	.30 (.22, .38)	.34 (.22, .46)	.30 (.16, .42)	.26 (.11, .41)	.49 (.31, .64)	.18 (.00, .35)	.27 (.03, .49)	.35 (.09, .57)
4	.40 (.33, .47)	.49 (.42, .55)	.45 (.39, .49)		.66 (.60, .72)	.44 (.35, .53)	.35 (.30, .41)	.39 (.29, .48)	.46 (.38, .53)	.31 (.23, .38)	.34 (.27, .41)	.27 (.18, .36)	.52 (.39, .63)	.26 (.15, .37)	.25 (.07, .41)	.22 (.08, .34)	.33 (.17, .48)	-.40 (-.77, .21)	NA (NA, NA)
3	.42 (.38, .46)	.44 (.40, .48)		.44 (.39, .49)	.54 (.49, .59)	.52 (.44, .60)	.46 (.42, .50)	.54 (.48, .60)	.44 (.37, .52)	.34 (.29, .40)	.39 (.33, .45)	.26 (.20, .32)	.29 (.17, .41)	.19 (.10, .29)	.23 (.11, .35)	.14 (.02, .25)	.22 (.10, .33)	.20 (-.01, .40)	.29 (.07, .48)
2	.46 (.42, .50)		.58 (.55, .62)	.52 (.45, .58)	.50 (.45, .55)	.46 (.36, .54)	.44 (.39, .50)	.41 (.33, .48)	.41 (.31, .49)	.32 (.26, .39)	.36 (.26, .45)	.27 (.19, .34)	.26 (.13, .38)	.13 (.01, .25)	.19 (.06, .32)	.36 (.19, .51)	.13 (-.03, .27)	.14 (-.12, .37)	.37 (.13, .56)
1		.54 (.50, .57)	.52 (.49, .55)	.51 (.44, .57)	.35 (.29, .40)	.34 (.25, .43)	.35 (.30, .40)	.46 (.41, .52)	.39 (.31, .47)	.30 (.25, .35)	.33 (.25, .41)	.23 (.17, .28)	.24 (.13, .34)	.27 (.17, .37)	.05 (-.07, .16)	.23 (.09, .36)	.17 (.04, .29)	.02 (-.18, .14)	.27 (.10, .42)

Supplementary table 5. Height correlations for males (upper triangular matrix) and females (lower triangular matrix) from 1 to 19 years of age.

	1	2	3	4	5	6	7	8	9	10	11	12	13	14	15	16	17	18	19
19	.55 (.47, .62)	.60 (.51, .69)	.64 (.55, .71)	.75 (.58, .86)	.59 (.49, .68)	.75 (.56, .86)	.66 (.58, .72)	.68 (.60, .74)	.82 (.75, .87)	.67 (.59, .73)	.66 (.59, .71)	.58 (.51, .65)	.65 (.58, .71)	.70 (.63, .76)	.67 (.59, .73)	.86 (.85, .88)	.92 (.92, .93)	.96 (.95, .96)	
18	.59 (.54, .63)	.70 (.65, .74)	.68 (.63, .72)	.75 (.70, .80)	.71 (.64, .76)	.74 (.68, .79)	.78 (.74, .81)	.77 (.73, .81)	.78 (.73, .82)	.78 (.75, .80)	.74 (.71, .76)	.66 (.60, .71)	.68 (.65, .71)	.76 (.71, .80)	.78 (.75, .80)	.88 (.86, .89)	.94 (.94, .95)		.98 (.97, .98)
17	.54 (.50, .59)	.63 (.58, .68)	.63 (.59, .67)	.67 (.62, .71)	.76 (.72, .80)	.84 (.79, .88)	.76 (.73, .78)	.77 (.73, .81)	.79 (.76, .82)	.80 (.78, .82)	.75 (.73, .77)	.73 (.69, .77)	.72 (.70, .74)	.85 (.82, .86)	.80 (.78, .82)	.93 (.93, .94)		.96 (.96, .97)	.97 (.97, .98)
16	.55 (.49, .60)	.68 (.63, .72)	.62 (.58, .66)	.63 (.59, .68)	.71 (.65, .76)	.82 (.78, .86)	.71 (.68, .74)	.69 (.65, .73)	.86 (.82, .89)	.76 (.73, .79)	.75 (.73, .78)	.77 (.73, .79)	.79 (.77, .81)	.88 (.84, .90)	.76 (.73, .79)		.96 (.96, .97)	.96 (.95, .96)	.95 (.94, .95)
15	.49 (.45, .53)	.61 (.57, .65)	.65 (.62, .69)	.65 (.61, .69)	.73 (.69, .76)	.82 (.78, .85)	.73 (.71, .76)	.79 (.76, .82)	.83 (.80, .85)	.83(.80, .85)	.82 (.81, .84)	.87 (.85, .89)	.95 (.94, .97)		.83 (.80, .85)	.90 (.87, .92)	.91 (.89, .92)	.94 (.93, .95)	.91 (.89, .93)
14	.46 (.42, .50)	.58 (.54, .62)	.57 (.54, .60)	.57 (.53, .61)	.68 (.64, .72)	.71 (.66, .75)	.69 (.67, .72)	.76 (.72, .79)	.78 (.75, .80)	.81 (.80, .83)	.83 (.82, .84)	.87 (.85, .89)		.89 (.84, .93)	.81 (.80, .83)	.88 (.87, .89)	.85 (.84, .87)	.86 (.85, .87)	.85 (.82, .88)
13	.45 (.41, .49)	.58 (.54, .62)	.64 (.61, .68)	.57 (.52, .62)	.71 (.67, .74)	.74 (.70, .78)	.73 (.71, .75)	.77 (.74, .79)	.78 (.75, .80)	.86 (.84, .88)	.87 (.85, .89)		.86 (.83, .87)	.83 (.81, .85)	.86 (.84, .88)	.69 (.65, .73)	.64 (.59, .69)	.58 (.47, .67)	.69 (.63, .73)
12	.52 (.50, .54)	.63 (.60, .65)	.65 (.63, .66)	.62 (.59, .65)	.75 (.73, .77)	.77 (.73, .80)	.79 (.78, .80)	.83 (.81, .84)	.88 (.86, .89)	.90 (.89, .91)		.86 (.84, .88)	.81 (.80, .82)	.76 (.74, .78)	.90 (.89, .91)	.67 (.64, .69)	.64 (.61, .67)	.68 (.65, .70)	.64 (.59, .68)
11	.51 (.48, .55)	.63 (.60, .67)	.67 (.65, .69)	.68 (.66, .70)	.78 (.75, .80)	.87 (.85, .88)	.81 (.80, .82)	.89 (.88, .90)	.91 (.91, .92)		.87 (.86, .88)	.84 (.82, .86)	.81 (.79, .82)	.74 (.70, .77)		.65 (.61, .69)	.68 (.65, .70)	.66 (.62, .71)	.62 (.54, .69)
10	.58 (.56, .60)	.69 (.67, .70)	.74 (.73, .75)	.69 (.67, .71)	.81 (.80, .83)	.88 (.86, .89)	.85 (.85, .86)	.90 (.89, .91)	.93 (.93, .94)	.90 (.90, .91)	.87 (.86, .88)	.84 (.82, .85)	.79 (.77, .81)	.78 (.76, .80)	.90 (.90, .91)	.71 (.68, .74)	.70 (.67, .73)	.67 (.63, .71)	.71 (.66, .75)

9	.54 (.50, .57)	.66 (.63, .69)	.74 (.72, .76)	.72 (.70, .75)	.80 (.77, .82)	.85 (.83, .87)	.83 (.82, .84)	.97 (.97, .97)		.88 (.87, .89)	.84 (.83, .86)	.84 (.81, .86)	.79 (.76, .81)	.73 (.68, .77)	.88 (.87, .89)	.76 (.68, .83)	.72 (.68, .76)	.74 (.61, .83)	.68 (.58, .76)
8	.58 (.56, .60)	.69 (.67, .71)	.78 (.76, .80)	.75 (.73, .78)	.83 (.81, .84)	.90 (.89, .91)	.93 (.92, .93)		.96 (.96, .97)	.85 (.84, .87)	.79 (.77, .81)	.80 (.78, .82)	.76 (.72, .79)	.81 (.77, .84)	.85 (.84, .87)	.70 (.65, .74)	.70 (.63, .75)	.77 (.71, .83)	.68 (.62, .74)
7	.61 (.60, .63)	.73 (.71, .74)	.74 (.73, .75)	.68 (.66, .70)	.83 (.82, .84)	.96 (.95, .96)		.92 (.92, .93)	.84 (.83, .85)	.78 (.76, .79)	.77 (.75, .78)	.77 (.75, .80)	.75 (.73, .77)	.77 (.75, .79)	.78 (.76, .79)	.65 (.62, .68)	.70 (.67, .72)	.75 (.70, .80)	.75 (.71, .79)
6	.57 (.54, .60)	.73 (.70, .75)	.85 (.83, .87)	.87 (.85, .88)	.93 (.92, .94)		.95 (.94, .96)	.82 (.80, .84)	.79 (.76, .81)	.83 (.80, .85)	.80 (.77, .83)	.76 (.71, .81)	.73 (.68, .78)	.81 (.75, .86)	.83 (.80, .85)	.72 (.64, .79)	.58 (.41, .71)	.62 (.35, .80)	.75 (.48, .89)
5	.61 (.59, .63)	.73 (.71, .74)	.82 (.81, .83)	.84 (.82, .85)		.95 (.94, .96)	.85 (.84, .86)	.81 (.79, .83)	.79 (.76, .81)	.72 (.68, .75)	.73 (.71, .75)	.72 (.68, .75)	.74 (.70, .77)	.71 (.67, .75)	.72 (.68, .75)	.70 (.65, .75)	.68 (.62, .72)	.67 (.60, .74)	.71 (.65, .77)
4	.57 (.54, .59)	.74 (.72, .76)	.73 (.71, .74)		.87 (.85, .88)	.84 (.82, .86)	.67 (.65, .69)	.73 (.70, .75)	.68 (.66, .71)	.61 (.59, .64)	.61 (.58, .64)	.63 (.58, .67)	.63 (.60, .66)	.65 (.60, .69)	.61 (.59, .64)	.63 (.58, .67)	.60 (.55, .65)	.67 (.46, .81)	.79 (.57, .91)
3	.71 (.70, .72)	.84 (.84, .85)		.71 (.69, .72)	.82 (.81, .83)	.85 (.84, .87)	.71 (.70, .73)	.77 (.75, .78)	.74 (.72, .76)	.61 (.58, .63)	.62 (.60, .64)	.61 (.57, .65)	.63 (.60, .66)	.69 (.66, .72)	.61 (.58, .63)	.58 (.54, .62)	.64 (.60, .67)	.69 (.62, .74)	.64 (.58, .70)
2	.76 (.75, .77)		.84 (.84, .85)	.76 (.74, .77)	.73 (.71, .74)	.74 (.71, .77)	.73 (.72, .75)	.69 (.67, .71)	.67 (.63, .70)	.59 (.55, .63)	.61 (.59, .63)	.58 (.54, .62)	.64 (.61, .67)	.67 (.63, .70)	.59 (.55, .63)	.64 (.59, .69)	.65 (.60, .69)	.61 (.53, .69)	.56 (.47, .63)
1		.75 (.74, .76)	.70 (.69, .71)	.59 (.56, .62)	.62 (.60, .64)	.54 (.50, .58)	.61 (.59, .63)	.56 (.54, .59)	.49 (.45, .53)	.45 (.41, .49)	.54 (.52, .56)	.52 (.48, .55)	.58 (.55, .61)	.64 (.61, .67)	.45 (.41, .49)	.62 (.57, .66)	.63 (.59, .66)	.62 (.57, .67)	.61 (.55, .66)

Supplementary table 6. Additive genetic correlations of height for males (upper triangular matrix) and females (lower triangular matrix) from 1 to 19 years of age.

	1	2	3	4	5	6	7	8	9	10	11	12	13	14	15	16	17	18	19
19	.60 (.50, .68)	.64 (.53, .73)	.67 (.57, .75)	NA (NA, NA)	.62 (.49, .72)	.70 (.42, .86)	.71 (.62, .79)	.72 (.63, .79)	.84 (.76, .90)	.78 (.71, .83)	.69 (.60, .77)	.69 (.62, .75)	.62 (.52, .71)	.70 (.60, .79)	.70 (.60, .78)	.90 (.89, .92)	.97 (.96, .98)	.96 (.95, .97)	
18	.60 (.53, .66)	.73 (.67, .78)	.70 (.63, .75)	.78 (.73, .83)	.73 (.66, .79)	.78 (.72, .83)	.79 (.75, .83)	.79 (.74, .83)	.79 (.73, .84)	.78 (.74, .81)	.80 (.76, .83)	.76 (.72, .78)	.68 (.60, .74)	.70 (.66, .73)	.79 (.74, .84)	.88 (.86, .90)	.97 (.96, .98)		.99 (.98, 1.00)
17	.58 (.52, .63)	.67 (.61, .72)	.67 (.62, .71)	.68 (.62, .73)	.78 (.73, .83)	.87 (.81, .91)	.77 (.74, .80)	.79 (.73, .84)	.82 (.78, .85)	.83 (.80, .85)	.81 (.79, .83)	.79 (.76, .82)	.78 (.73, .83)	.75 (.72, .78)	.87 (.84, .89)	.96 (.96, .97)		1.00 (.99, 1.00)	.99 (.99, .99)
16	.58 (.52, .65)	.71 (.64, .76)	.67 (.62, .72)	.70 (.64, .75)	.74 (.66, .80)	.84 (.79, .88)	.74 (.70, .77)	.72 (.67, .77)	.86 (.80, .90)	.80 (.77, .83)	.85 (.80, .89)	.78 (.75, .81)	.82 (.78, .85)	.81 (.79, .84)	.86 (.82, .90)		.99 (.99, .99)	.97 (.97, .98)	.97 (.97, .98)
15	.52 (.47, .57)	.63 (.58, .68)	.68 (.63, .72)	.66 (.61, .71)	.73 (.69, .77)	.86 (.82, .89)	.77 (.74, .80)	.80 (.76, .84)	.84 (.80, .87)	.83 (.80, .85)	.91 (.88, .93)	.85 (.83, .87)	.89 (.87, .91)	.96 (.95, .98)		.96 (.93, NA)	.96 (.95, .97)	.96 (.94, .97)	.94 (.91, .96)
14	.51 (.45, .56)	.61 (.56, .66)	.61 (.57, .65)	.61 (.57, .65)	.71 (.67, .76)	.75 (.70, .80)	.73 (.70, .75)	.77 (.73, .81)	.81 (.78, .83)	.79 (.76, .82)	.85 (.84, .87)	.86 (.85, .87)	.88 (.86, .91)		.91 (.83, .95)	.93 (.91, .94)	.89 (.88, .91)	.88 (.86, .90)	.90 (.85, .93)
13	.49 (.44, .54)	.60 (.56, .65)	.65 (.61, .69)	.62 (.57, .67)	.73 (.69, .76)	.77 (.72, .81)	.73 (.70, .76)	.78 (.75, .81)	.79 (.76, .82)	.82 (.79, .84)	.86 (.84, .88)	.90 (.87, .92)		.91 (.88, .93)	.84 (.81, .87)	.76 (.71, .81)	.68 (.61, .75)	.55 (.39, .68)	.70 (.63, .77)
12	.55 (.53, .58)	.66 (.63, .68)	.67 (.64, .69)	.64 (.61, .68)	.77 (.74, .79)	.80 (.76, .83)	.82 (.80, .83)	.84 (.82, .86)	.89 (.87, .90)	.89 (.88, .90)	.91 (.90, .92)		.94 (.91, .96)	.84 (.83, .86)	.77 (.74, .80)	.72 (.68, .75)	.67 (.63, .71)	.69 (.65, .72)	.67 (.60, .72)
11	.55 (.51, .59)	.68 (.64, .72)	.72 (.69, .74)	.71 (.68, .73)	.80 (.77, .83)	.89 (.87, .91)	.83 (.82, .84)	.90 (.88, .91)	.92 (.92, .93)	.92 (.91, .93)		.88 (.87, .89)	.85 (.82, .87)	.84 (.82, .85)	.76 (.71, .79)	.72 (.67, .77)	.70 (.66, .73)	.68 (.62, .73)	.63 (.54, .71)
10	.61 (.58, .63)	.71 (.69, .73)	.76 (.75, .78)	.73 (.71, .75)	.83 (.82, .85)	.89 (.87, .91)	.87 (.86, .88)	.91 (.90, .92)	.94 (.93, .95)		.90 (.89, .91)	.87 (.86, .88)	0 .85 (.82, .87)	.84 (.81, .86)	.80 (.77, .82)	.74 (.71, .77)	.72 (.68, .76)	.69 (.63, .74)	.74 (.67, .79)

9	.56 (.52, .60)	.68 (.64, .72)	.75 (.73, .78)	.75 (.73, .78)	.83 (.80, .85)	.87 (.85, .89)	.85 (.83, .86)	.98 (.98, .98)		.93 (.93, .94)	.89 (.88, .91)	.86 (.84, .88)	.86 (.83, .88)	.83 (.80, .85)	.76 (.70, .81)	.81 (.71, .87)	.75 (.70, .80)	.74 (.57, .85)	.69 (.56, .78)
8	.61 (.58, .64)	.72 (.69, .75)	.80 (.78, .82)	.78 (.76, .81)	.84 (.82, .86)	.90 (.89, .92)	.94 (.93, .94)		.98 (.97, .98)	.87 (.86, .89)	.86 (.85, .88)	.81 (.79, .83)	.82 (.80, .85)	.78 (.74, .82)	.82 (.78, .86)	.70 (.65, .75)	.69 (.60, .76)	.78 (.70, .84)	.70 (.61, .77)
7	.64 (.62, .66)	.75 (.73, .77)	.77 (.76, .79)	.72 (.70, .74)	.84 (.83, .85)	.97 (.97, .98)		.94 (.93, .94)	.85 (.83, .86)	.86 (.85, .87)	.79 (.77, .80)	.78 (.77, .80)	.79 (.77, .82)	.78 (.76, .81)	.78 (.75, .81)	.69 (.65, .73)	.74 (.71, .77)	.77 (.71, .83)	.75 (.69, .80)
6	.62 (.58, .66)	.77 (.74, .80)	.88 (.87, .90)	.89 (.87, .90)	.95 (.94, .96)		.97 (.96, .98)	.84 (.82, .86)	.82 (.79, .84)	.88 (.85, .89)	.85 (.83, .88)	.81 (.77, .84)	.76 (.70, .82)	.73 (.66, .78)	.83 (.76, .88)	.64 (.49, .75)	.52 (.29, .69)	.40 (.02, .69)	.76 (.34, 1.00)
5	.64 (.62, .66)	.75 (.74, .77)	.85 (.84, .86)	.87 (.85, .89)		.95 (.94, .96)	.86 (.85, .87)	.83 (.81, .85)	.81 (.78, .84)	.82 (.80, .83)	.74 (.69, .77)	.74 (.72, .77)	.76 (.72, .80)	.78 (.74, .82)	.75 (.70, .79)	.70 (.63, .76)	.70 (.63, .76)	.68 (.58, .77)	.67 (.58, .74)
4	.60 (.57, .64)	.77 (.75, .79)	.76 (.74, .77)		.88 (.87, .90)	.86 (.84, .88)	.71 (.69, .72)	.76 (.73, .79)	.73 (.70, .76)	.67 (.65, .70)	.65 (.62, .67)	.65 (.62, .68)	.63 (.58, .69)	.67 (.63, .71)	.66 (.60, .71)	.65 (.60, .70)	.65 (.58, .70)	.54 (.21, .76)	NA (NA, NA)
3	.74 (.73, .76)	.87 (.87, .88)		.74 (.72, .76)	.84 (.83, .85)	.87 (.85, .89)	.74 (.73, .75)	.78 (.76, .80)	.77 (.74, .79)	.73 (.71, .74)	.64 (.61, .66)	.65 (.63, .67)	.63 (.58, .67)	.67 (.63, .70)	.70 (.66, .73)	.65 (.59, .69)	.68 (.63, .71)	.69 (.61, .76)	.66 (.57, .72)
2	.79 (.78, .80)		.87 (.86, .87)	.78 (.75, .80)	.75 (.74, .77)	.76 (.72, .79)	.74 (.72, .76)	.72 (.69, .74)	.69 (.65, .73)	.68 (.66, .70)	.62 (.57, .67)	.63 (.61, .66)	.61 (.55, .66)	.66 (.61, .71)	.68 (.64, .73)	.67 (.60, .73)	.66 (.60, .72)	.60 (.48, .70)	.59 (.49, .69)
1		.79 (.78, .80)	.73 (.72, .75)	.61 (.58, .64)	.65 (.63, .67)	.57 (.52, .61)	.63 (.61, .65)	.60 (.57, .63)	.52 (.47, .56)	.59 (.57, .61)	.49 (.44, .54)	.57 (.54, .60)	.53 (.48, .58)	.60 (.56, .65)	.66 (.62, .70)	.63 (.57, .68)	.65 (.59, .69)	.62 (.55, .68)	.63 (.55, .69)

Supplementary table 7. Unique environmental correlations of height for males (upper triangular matrix) and females (lower triangular matrix) from 1 to 19 years of age.

	1	2	3	4	5	6	7	8	9	10	11	12	13	14	15	16	17	18	19
19	.31 (.09, .50)	.16 (-.16, .45)	.48 (.23, .67)	NA (NA, NA)	.67 (.48, .80)	.64 (.11, .88)	.44 (.22, .61)	.09 (-.20, .36)	.62 (.37, .78)	.41 (.17, .60)	.62 (.44, .75)	.04 (-.18, .26)	.28 (.07, .48)	.25 (.00, .46)	.60 (.40, .74)	.50 (.40, .59)	.54 (.45, .63)	.80 (.71, .86)	
18	.42 (.29, .53)	.41 (.26, .54)	.44 (.29, .58)	.48 (.32, .61)	.39 (.18, .56)	.53 (.37, .66)	.44 (.30, .56)	.42 (.24, .57)	.52 (.35, .66)	.59 (.49, .68)	.42 (.28, .54)	.46 (.36, .55)	.49 (.34, .62)	.40 (.30, .49)	.20 (.01, .38)	.65 (.56, .73)	.70 (.61, .77)		.91 (.87, .94)
17	.41 (.29, .52)	.26 (.10, .42)	.30 (.17, .42)	.42 (.25, .56)	.54 (.37, .67)	.28 (-.03, .55)	.50 (.40, .59)	.42 (.22, .59)	.49 (.37, .60)	.51 (.39, .61)	.54 (.45, .62)	.34 (.24, .44)	.43 (.29, .56)	.31 (.21, .41)	.54 (.42, .64)	.72 (.67, .76)		.73 (.67, .78)	.83 (.79, .86)
16	.31 (.17, .44)	.45 (.31, .58)	.20 (.06, .33)	.10 (-.06, .25)	.46 (.24, .63)	.46 (.25, .63)	.36 (.25, .46)	.34 (.20, .48)	.67 (.46, .80)	.55 (.46, .63)	.34 (.18, .48)	.40 (.30, .49)	.40 (.27, .52)	.54 (.45, .62)	.57 (.39, .71)		.76 (.73, .79)	.78 (.73, .83)	.71 (.66, .75)
15	.29 (.18, .40)	.37 (.25, .48)	.40 (.28, .50)	.47 (.34, .58)	.48 (.33, .60)	.36 (.17, .52)	.38 (.29, .47)	.53 (.40, .64)	.69 (.59, .76)	.41 (.31, .50)	.26 (.14, .39)	.44 (.35, .51)	.58 (.47, .66)	.81 (.69, .88)		.48 (.25, .66)	.51 (.42, .60)	.73 (.63, .81)	.68 (.57, .77)
14	.18 (.07, .29)	.33 (.20, .44)	.30 (.20, .40)	.30 (.18, .41)	.29 (.14, .43)	.47 (.29, .61)	.37 (.28, .45)	.48 (.35, .59)	.55 (.46, .64)	.55 (.47, .63)	.40 (.33, .47)	.49 (.43, .55)	.56 (.43, .66)		.87 (.75, .94)	.56 (.48, .63)	.57 (.50, .63)	.67 (.61, .73)	.53 (.36, .67)
13	.30 (.19, .39)	.37 (.26, .47)	.51 (.41, .61)	.30 (.15, .43)	.48 (.37, .58)	.44 (.28, .58)	.57 (.49, .64)	.57 (.48, .65)	.46 (.35, .56)	.66 (.58, .72)	.79 (.74, .84)	.58 (.47, .68)		.47 (.35, .58)	.70 (.62, .77)	.32 (.18, .46)	.39 (.24, .52)	.65 (.45, .78)	.40 (.22, .56)
12	.32 (.26, .38)	.39 (.32, .45)	.41 (.35, .47)	.47 (.39, .55)	.51 (.44, .58)	.56 (.46, .65)	.49 (.44, .54)	.66 (.61, .71)	.72 (.67, .77)	.67 (.63, .70)	.77 (.72, .80)		.27 (.11, .42)	.56 (.50, .60)	.60 (.53, .66)	.27 (.18, .37)	.33 (.23, .43)	.52 (.43, .59)	.33 (.18, .46)
11	.30 (.21, .38)	.26 (.15, .36)	.33 (.26, .40)	.47 (.40, .53)	.61 (.52, .69)	.61 (.52, .68)	.62 (.58, .66)	.75 (.71, .79)	.78 (.74, .81)	.71 (.66, .75)		.67 (.62, .72)	.70 (.63, .77)	.54 (.48, .60)	.51 (.39, .62)	.32 (.18, .44)	.44 (.35, .53)	.51 (.37, .63)	.65 (.49, .77)
10	.46 (.41, .51)	.50 (.45, .56)	.55 (.50, .59)	.52 (.46, .58)	.67 (.62, .72)	.74 (.69, .79)	.61 (.57, .64)	.76 (.73, .80)	.77 (.73, .81)		.79 (.76, .82)	.75 (.72, .78)	.67 (.59, .74)	.45 (.36, .53)	.61 (.53, .68)	.53 (.44, .61)	.47 (.37, .57)	.56 (.44, .66)	.41 (.24, .56)

9	.44 (.37, .51)	.38 (.28, .47)	.61 (.54, .66)	.51 (.44, .58)	.57 (.49, .64)	.72 (.65, .77)	.73 (.70, .77)	.83 (.79, .85)		.77 (.73, .80)	.74 (.70, .77)	.71 (.65, .76)	.63 (.53, .71)	.54 (.45, .62)	.53 (.38, .66)	.64 (.39, .81)	.52 (.40, .63)	.83 (.58, .93)	.59 (.31, .78)
8	.43 (.37, .49)	.46 (.39, .53)	.56 (.50, .62)	.58 (.50, .65)	.68 (.62, .73)	.76 (.71, .80)	.85 (.82, .87)		.83 (.80, .85)	.78 (.75, .81)	.73 (.68, .77)	.69 (.64, .73)	.58 (.48, .66)	.65 (.55, .73)	.57 (.42, .69)	.68 (.58, .77)	.62 (.46, .74)	.69 (.51, .81)	.52 (.34, .66)
7	.50 (.46, .54)	.54 (.49, .59)	.50 (.46, .54)	.50 (.45, .55)	.73 (.69, .77)	.73 (.67, .77)		.82 (.79, .85)	.75 (.71, .78)	.69 (.66, .72)	.61 (.57, .65)	.61 (.57, .65)	.54 (.45, .62)	.54 (.47, .60)	.65 (.58, .71)	.47 (.38, .55)	.45 (.36, .54)	.58 (.41, .71)	.46 (.28, .61)
6	.44 (.36, .51)	.53 (.45, .60)	.52 (.44, .60)	.68 (.62, .73)	.73 (.67, .79)		.76 (.71, .80)	.72 (.67, .77)	.64 (.57, .71)	.63 (.56, .69)	.57 (.48, .65)	.53 (.41, .62)	.70 (.57, .80)	.71 (.59, .80)	.47 (.18, .68)	.78 (.62, .88)	.82 (.58, .92)	.64 (-.01, .91)	.81 (.27, NA)
5	.45 (.39, .50)	.53 (.48, .58)	.63 (.58, .67)	.70 (.64, .74)		.86 (.81, .89)	.74 (.70, .77)	.71 (.65, .76)	.62 (.54, .69)	.58 (.53, .64)	.66 (.58, .73)	.49 (.41, .55)	.37 (.24, .48)	.44 (.32, .55)	.46 (.33, .58)	.67 (.53, .77)	.34 (.16, .49)	.51 (.30, .68)	.55 (.32, .72)
4	.46 (.39, .52)	.56 (.50, .61)	.61 (.57, .65)		.78 (.73, .82)	.61 (.53, .67)	.52 (.47, .57)	.60 (.52, .66)	.49 (.41, .56)	.44 (.38, .51)	.50 (.43, .56)	.33 (.24, .41)	.49 (.35, .61)	.39 (.29, .48)	.51 (.36, .63)	.52 (.41, .62)	.31 (.15, .46)	.84 (.50, .95)	NA (NA, NA)
3	.52 (.49, .56)	.58 (.55, .62)		.59 (.55, .63)	.73 (.70, .76)	.66 (.59, .71)	.57 (.54, .61)	.68 (.63, .72)	.56 (.49, .62)	.55 (.51, .60)	.43 (.37, .49)	.42 (.36, .47)	.47 (.37, .57)	.42 (.34, .50)	.62 (.54, .69)	.21 (.09, .32)	.43 (.32, .52)	.67 (.53, .77)	.30 (.08, .49)
2	.56 (.53, .59)		.67 (.64, .69)	.60 (.54, .65)	.62 (.57, .66)	.58 (.50, .65)	.65 (.61, .69)	.55 (.48, .61)	.50 (.41, .57)	.47 (.41, .53)	.44 (.34, .52)	.42 (.35, .48)	.44 (.32, .54)	.50 (.40, .58)	.45 (.33, .55)	.52 (.37, .64)	.45 (.32, .56)	.69 (.53, .80)	.29 (.05, .50)
1		.54 (.51, .57)	.53 (.49, .56)	.48 (.41, .54)	.46 (.41, .51)	.35 (.27, .44)	.48 (.43, .52)	.39 (.33, .45)	.38 (.30, .45)	.44 (.39, .48)	.26 (.18, .34)	.32 (.27, .38)	.36 (.26, .45)	.39 (.30, .48)	.47 (.38, .55)	.44 (.32, .55)	.46 (.35, .55)	.56 (.44, .67)	.29 (.13, .44)

Supplementary table 8. Additive genetic correlations of BMI for males (upper triangular matrix) and females (lower triangular matrix) from 1 to 19 years of age under ACE model.

	1	2	3	4	5	6	7	8	9	10	11	12	13	14	15	16	17	18	19
19	.13 (-1.00, 1.00)	.62 (.62, .92)	.66 (-.40, NA)	NA (NA, NA)	.59 (.59, NA)	NA (NA, NA)	.48 (.48, .70)	.49 (.18, .71)	NA (NA, NA)	.66 (.66, .88)	.96 (.74, 1.00)	.69 (.69, 1.00)	.74 (.51, .91)	.91 (.71, 1.00)	.80 (.80, .98)	.96 (.89, NA)	.99 (.94, NA)	1.00 (.92, NA)	
18	.32 (.32, .51)	.48 (.24, .70)	.37 (.19, .61)	.58 (NA, NA)	.53 (.50, NA)	.61 (NA, NA)	.63 (.63, .80)	.72 (.59, .86)	.78 (NA, NA)	.71 (NA, .77)	.73 (.69, .82)	.73 (.66, .81)	.77 (.77, .85)	.80 (.76, .84)	.88 (NA, .93)	.87 (.84, .92)	.93 (.90, .97)		.93 (.93, 1.00)
17	.34 (.19, .51)	.36 (.14, .54)	.37 (.36, .61)	.41 (.26, .58)	.64 (.64, .87)	.58 (.43, .73)	.58 (.48, .68)	.74 (.60, .88)	.86 (.70, 1.00)	.76 (.69, .85)	.71 (.64, .78)	.74 (.68, .83)	.94 (.82, 1.00)	.87 (.83, .90)	.94 (.87, NA)	.97 (.94, 1.00)		.94 (.91, 1.00)	.96 (NA, NA)
16	.28 (.21, .48)	.35 (.35, .51)	.43 (.43, .65)	.33 (.33, .57)	.40 (.38, .65)	.78 (NA, 1.00)	.60 (.42, .75)	.75 (.75, .94)	.82 (.64, NA)	.73 (.58, .83)	.82 (.62, 1.00)	.77 (.66, .86)	1.00 (.83, NA)	.90 (.87, .94)	.86 (NA, .94)		.95 (.93, .96)	.95 (.91, 1.00)	.90 (.88, .93)
15	.38 (.15, .52)	.40 (.25, .53)	.38 (.38, .57)	.61 (.45, .79)	.68 (.68, .93)	.78 (NA, 1.00)	.65 (.54, .77)	.69 (.69, .82)	.74 (.74, .82)	.80 (.72, .87)	.89 (.82, .97)	.82 (.78, .86)	.86 (.77, .93)	.99 (.97, NA)		.91 (NA, .96)	.85 (.80, .90)	.90 (.87, NA)	.85 (.77, .92)
14	.17 (.03, .31)	.14 (-.07, .15)	.40 (.27, .60)	.43 (.43, .60)	.40 (.23, NA)	.63 (.42, NA)	.66 (.55, .76)	.74 (.74, NA)	.74 (.66, .81)	.77 (.68, .86)	.87 (.81, .92)	.95 (.90, .99)	.77 (.69, .84)		.95 (NA, NA)	.96 (.91, 1.00)	.84 (.80, .91)	.91 (.91, 1.00)	.87 (.78, .97)
13	.36 (.21, .50)	.34 (NA, .45)	.46 (.46, .62)	.34 (.11, .45)	.86 (NA, 1.00)	.62 (.49, .72)	.65 (.59, .73)	.74 (NA, NA)	.90 (.81, .99)	.85 (.78, .90)	.85 (.80, .89)	.87 (NA, NA)		1.00 (.92, NA)	.93 (.85, 1.00)	.89 (.84, .97)	.90 (.90, 1.00)	.62 (NA, .92)	.65 (.65, .77)
12	.27 (.17, .35)	.41 (.31, NA)	.43 (.35, .51)	.50 (.37, .63)	.60 (NA, NA)	.46 (.32, .64)	.75 (.69, .81)	.67 (.59, .75)	.91 (.86, NA)	.87 (.83, .90)	.81 (.76, .85)		1.00 (.95, NA)	.90 (.85, .94)	.82 (.77, .88)	.80 (.71, .87)	.78 (.69, .85)	.72 (.65, .79)	.75 (.66, .89)
11	.32 (.18, .54)	.37 (.26, .47)	.41 (.32, .51)	.54 (.43, .63)	.60 (.51, NA)	.68 (.63, .77)	.75 (.70, .81)	.80 (.75, .83)	.90 (.85, .95)	.82 (.78, .85)		.87 (.85, .88)	.93 (.85, NA)	.82 (.77, .88)	.81 (.75, .87)	.85 (.70, .98)	.75 (.62, .83)	.75 (.61, .87)	.79 (NA, NA)
10	.30 (.22, .38)	.34 (NA, .50)	.51 (.43, .60)	.61 (.50, .71)	.74 (.66, .81)	.86 (.75, .95)	.83 (.78, .87)	.84 (.79, .89)	.95 (.92, .98)		.82 (.78, .85)	.82 (.79, .84)	.83 (.78, .88)	.75 (.62, .83)	.77 (.72, .84)	.75 (NA, .82)	.74 (.63, .83)	.58 (.46, .95)	.66 (NA, NA)

9	.38 (.28, NA)	.42 (.27, .59)	.54 (.43, .65)	.50 (.35, .64)	.81 (.66, .90)	.90 (.81, .97)	.76 (.69, .81)	.94 (.93, .97)		.92 (.90, .93)	.91 (.86, .94)	.95 (.88, 1.00)	.86 (.79, .93)	.78 (.68, .87)	.77 (NA, .84)	.99 (NA, NA)	.71 (.71, .85)	.69 (-1.00, 1.00)	NA (NA, NA)
8	.44 (.34, .54)	.57 (.48, .65)	.63 (.57, .70)	.58 (.49, .66)	.67 (.67, .79)	.95 (.89, 1.00)	.97 (.94, NA)		.97 (.93, NA)	.89 (.85, NA)	.79 (.73, .84)	.80 (.73, .86)	.96 (.86, NA)	.93 (.80, NA)	.77 (.65, .86)	.73 (.56, .88)	.63 (.52, .81)	.73 (.73, 1.00)	.85 (NA, NA)
7	.42 (.35, .48)	.54 (.46, .60)	.65 (.59, .71)	.58 (.50, .65)	.87 (.80, .92)	.99 (.95, NA)		.92 (NA, .96)	.84 (.76, .88)	.81 (.77, .84)	.70 (.63, .75)	.71 (.67, .76)	.78 (.70, .85)	.64 (.54, .75)	.69 (.59, .78)	.64 (.51, .77)	.74 (.62, .85)	.66 (.66, .83)	.45 (.45, .60)
6	.42 (.27, .55)	.74 (.62, .85)	.78 (NA, NA)	0 .89 (NA, NA)	1.00 (.97, NA)		.98 (NA, 1.00)	.92 (.86, .98)	.92 (.84, .98)	.84 (.80, .91)	.76 (NA, .85)	.72 (.61, NA)	.79 (.70, .91)	.50 (NA, .63)	.68 (.49, .85)	.79 (.69, 1.00)	.73 (.47, NA)	-1 (NA, NA)	NA (NA, NA)
5	.48 (NA, NA)	.60 (.53, .66)	.68 (.61, .74)	.72 (NA, .80)		.99 (NA, NA)	.75 (.69, .80)	.87 (.79, .94)	.71 (.52, .80)	.67 (.60, .73)	.60 (.60, .75)	.63 (NA, NA)	.48 (NA, NA)	.56 (-.24, .70)	.56 (.39, .69)	.52 (.52, .73)	.79 (NA, 1.00)	.10 (-1.00, 1.00)	.19 (-.28, .19)
4	.41 (.29, .52)	.73 (.66, .79)	.77 (.71, .84)		.82 (.80, .91)	.99 (.94, 1.00)	.68 (.60, .76)	.58 (.58, .78)	.57 (.45, .67)	.47 (.38, .56)	.51 (.41, .59)	.47 (.37, .57)	.47 (.24, .65)	.50 (.36, .66)	.46 (.46, .63)	.72 (.72, NA)	.37 (.37, .56)	.55 (-1.00, 1.00)	NA (NA, NA)
3	.67 (.62, .72)	.85 (.81, .88)		.71 (.63, .78)	.74 (.69, .78)	.77 (.66, NA)	.58 (.52, .64)	.68 (.59, .74)	.74 (.64, .83)	.49 (.42, .55)	.42 (.33, .52)	.50 (.42, .57)	.49 (.34, .64)	.35 (.25, .46)	.42 (.32, .55)	.52 (.52, .79)	.44 (.31, .60)	.38 (.26, .58)	.38 (.04, .57)
2	.78 (.73, .83)		.78 (.74, .81)	.76 (.67, .84)	.64 (.56, .71)	.64 (.54, .74)	.54 (.44, .62)	.57 (.38, .68)	.51 (.39, .64)	.32 (NA, .39)	.33 (.33, .56)	.36 (.24, NA)	.31 (.26, .48)	.24 (.24, .49)	.38 (.38, .59)	.36 (NA, .58)	.53 (.15, .74)	.34 (NA, .76)	.29 (-.93, NA)
1		.78 (.74, .82)	.68 (.63, .73)	.55 (.44, .65)	.59 (NA, .70)	.51 (.36, .66)	.43 (.34, .51)	.39 (.27, .51)	.43 (.42, .59)	.35 (.27, .44)	.37 (.21, .53)	.34 (.25, .43)	.32 (.18, .47)	.16 (.03, .35)	.39 (.28, .53)	.15 (-.10, .40)	.32 (.16, .46)	.32 (NA, .45)	.13 (.05, .37)

Supplementary table 9. Additive genetic correlations of height for males (upper triangular matrix) and females (lower triangular matrix) from 1 to 19 years of age under ACE model.

	1	2	3	4	5	6	7	8	9	10	11	12	13	14	15	16	17	18	19
19	.64 (.42, .86)	.69 (-.05, NA)	.71 (.71, NA)	NA (NA, NA)	.85 (.28, NA)	NA (NA, NA)	.89 (.62, 1.00)	.79 (.59, NA)	.86 (.51, 1.00)	.85 (.63, NA)	.58 (.20, NA)	.66 (.54, .75)	.68 (.39, .89)	.56 (.25, .86)	.72 (.61, NA)	.91 (.88, .94)	.96 (.95, .98)	.97 (NA, NA)	
18	.70 (.54, .83)	.81 (.66, .91)	.76 (.57, .88)	.80 (NA, .88)	.81 (.61, NA)	.82 (.75, NA)	.80 (.72, .86)	.81 (.72, NA)	.79 (NA, NA)	.76 (.68, .82)	.77 (.70, .86)	.74 (.69, .78)	.68 (.58, .76)	.63 (.56, .69)	.84 (.77, .90)	.84 (.79, .87)	.96 (.94, .97)		1.00 (NA, NA)
17	.71 (.55, .86)	.67 (.54, .79)	.65 (.50, .76)	.68 (.54, .78)	.79 (.66, .88)	.86 (NA, .93)	.78 (.72, .81)	.73 (.62, .82)	.81 (.71, .89)	.85 (NA, NA)	.78 (.73, .82)	.76 (.71, .80)	.81 (.69, NA)	.78 (.75, .81)	.87 (.82, .91)	.96 (NA, .98)		.99 (.99, 1.00)	.99 (.98, 1.00)
16	.61 (.40, .80)	.62 (.41, .79)	.73 (.58, .88)	.77 (.62, .91)	.83 (.65, .93)	.87 (NA, NA)	.77 (.70, .84)	.82 (.71, .89)	.84 (.74, .91)	.77 (.71, .82)	.90 (.81, NA)	.78 (.75, .81)	.85 (.76, .92)	.82 (.80, .85)	.80 (NA, NA)		.99 (.98, .99)	.97 (.96, .98)	.97 (.96, .98)
15	.49 (.37, .62)	.61 (.47, .72)	.71 (.60, .79)	.65 (.54, .75)	.74 (.65, .81)	.86 (.80, .92)	.82 (.76, .89)	.79 (.68, .86)	.84 (.78, NA)	.81 (.77, .85)	.94 (NA, NA)	.84 (.81, .86)	.91 (.86, .95)	.97 (NA, .99)		.97 (.91, NA)	.95 (.92, NA)	.96 (.93, .99)	.97 (.90, 1.00)
14	.59 (.42, .87)	.66 (.53, .76)	.63 (.54, .70)	.62 (.54, .71)	.76 (.66, .84)	.79 (.71, .86)	.80 (.74, .85)	.80 (.68, .86)	.86 (.81, .90)	.79 (.76, .83)	.86 (.81, .90)	.91 (.88, .94)	.89 (.86, .92)		1.00 (.85, NA)	.94 (.89, .99)	.88 (.84, .93)	.87 (.83, .91)	.94 (.82, 1.00)
13	.55 (.43, .68)	.67 (.57, .76)	.65 (.53, .74)	.70 (.57, .82)	.78 (NA, .86)	.83 (.73, .90)	.75 (.68, .81)	.83 (.76, .89)	.87 (.80, .91)	.83 (.78, .88)	.88 (.83, .91)	NA (NA, NA)		1.00 (.93, NA)	.82 (.73, .89)	.73 (.63, .84)	.62 (.39, NA)	.49 (.08, .71)	.70 (.44, .86)
12	.53 (.46, .60)	.68 (.61, .74)	.71 (.66, .76)	.65 (.58, .71)	.76 (.71, .81)	.86 (.80, .90)	.91 (.88, .93)	.89 (.86, .92)	.91 (.88, .94)	.93 (.91, .94)	.93 (.90, .95)		NA (NA, NA)	.89 (.86, .92)	.79 (.73, .84)	.71 (.65, .78)	.67 (.61, .72)	.71 (.65, .77)	.68 (.51, .83)
11	.62 (.51, .73)	.80 (.68, .89)	.78 (.73, .82)	.75 (.70, .80)	.88 (.81, .93)	.93 (.88, .96)	.84 (.81, .87)	.91 (.88, .93)	.96 (.95, .98)	.91 (.88, .92)		.89 (.86, .91)	.84 (.80, .87)	.86 (.82, .89)	.79 (NA, .86)	.88 (.72, NA)	.63 (.56, .72)	.63 (.49, .79)	.69 (NA, NA)
10	.61 (.55, .66)	.77 (.72, .82)	.80 (.76, .83)	.82 (.77, .87)	.90 (.87, .92)	.93 (.91, .96)	.94 (.92, .96)	.97 (.96, .98)	.94 (.93, .95)		.89 (.87, .91)	.87 (.85, .88)	.85 (.81, .87)	.88 (.80, .95)	.73 (.66, .82)	.75 (.67, .81)	.74 (NA, NA)	.62 (.46, .76)	.81 (.68, .91)

9	.58 (.44, .69)	.77 (.69, .84)	.87 (.82, .92)	.83 (.77, .87)	.93 (.86, .99)	.98 (.95, 1.00)	.92 (.90, .94)	1.00 (.99, 1.00)		.95 (.93, .97)	.91 (.88, .93)	.91 (.86, .94)	.92 (.87, .97)	.97 (.91, 1.00)	.86 (.73, NA)	.90 (.71, NA)	.78 (.55, .89)	.95 (.79, NA)	.76 (.54, .89)
8	.66 (.59, .73)	.84 (.78, .89)	.89 (.86, .92)	.92 (.87, .95)	.96 (.93, .98)	.95 (.93, .97)	.98 (.97, NA)		1.00 (.99, NA)	.90 (.87, .91)	.86 (.82, .90)	.84 (.80, .88)	.82 (.75, .88)	.90 (.82, .96)	.81 (.65, .91)	.82 (.71, .89)	.51 (.05, .76)	.63 (NA, .86)	.62 (.33, .83)
7	.66 (.60, .71)	.80 (.75, .83)	.91 (.89, .93)	.85 (.81, .88)	.91 (.88, .93)	1.00 (NA, NA)		.96 (NA, .98)	.93 (.91, .94)	.90 (.88, .92)	.81 (.78, .84)	.78 (.74, .81)	.79 (.74, .82)	.79 (.74, .84)	.76 (.68, .82)	.77 (.69, .84)	.74 (.65, .82)	.78 (.64, .88)	.79 (.67, .88)
6	.76 (.65, .85)	.86 (.79, .92)	.95 (.91, 1.00)	.99 (NA, NA)	1.00 (.98, NA)		1.00 (NA, NA)	.99 (.97, NA)	.94 (.90, .97)	.94 (.90, .97)	.89 (.84, .94)	.85 (.75, .92)	.78 (.67, .87)	.71 (.51, .84)	.82 (.69, .91)	.62 (.29, NA)	.49 (NA, .73)	.39 (-.19, NA)	NA (NA, NA)
5	.74 (.68, .79)	.86 (.83, .89)	.93 (.91, .95)	.97 (.94, NA)		.96 (NA, .97)	.90 (.87, .92)	.89 (.85, .93)	.88 (.83, .92)	.87 (.83, .90)	.72 (.62, .80)	.76 (.70, .80)	.78 (.69, .87)	.83 (.73, .91)	.78 (.62, .89)	.73 (.63, .81)	.73 (.52, .87)	.69 (.41, .86)	.70 (NA, NA)
4	.75 (.67, .82)	.92 (.88, .96)	.91 (.88, .94)		.98 (.96, .99)	.95 (NA, NA)	.83 (.80, .87)	.86 (.78, .92)	.88 (.83, .92)	.73 (.67, .78)	.73 (.67, .78)	.74 (.65, .80)	.69 (.60, .76)	.82 (.73, .90)	.73 (.59, .83)	.69 (.59, .78)	.63 (.46, .77)	.25 (-1.00, 1.00)	NA (NA, NA)
3	.85 (.82, .89)	.92 (.91, .94)		.86 (.82, .89)	.90 (.87, .92)	.93 (.89, .97)	.83 (.80, .86)	.88 (.84, .91)	.82 (.76, .86)	.75 (.71, .79)	.71 (.64, .76)	.66 (.61, .72)	.63 (.51, .72)	.69 (.60, .78)	.66 (.53, .75)	.88 (.76, NA)	.68 (.59, .75)	.71 (.58, .82)	.66 (.54, .78)
2	.88 (.86, .91)		.89 (.87, .91)	.88 (.84, .92)	.84 (.80, .87)	.81 (.73, .89)	.78 (.74, .82)	.73 (.67, .79)	.79 (.69, .86)	.75 (.69, .79)	.66 (.55, .77)	.68 (.61, .75)	.68 (.53, .87)	.66 (.56, .75)	.66 (.53, .76)	.70 (.55, .80)	.61 (.40, .75)	.54 (.33, .68)	.55 (.32, .77)
1		.90 (.87, .93)	.85 (.81, .88)	.73 (.65, .80)	.75 (.70, .80)	.73(.63, .82)	.73 (.68, .78)	.68 (.60, .76)	.61 (.52, .71)	.68 (.62, .74)	.57 (.47, .67)	.62 (.55, .70)	.50 (.38, .63)	.53 (.37, .70)	.63 (.51, .74)	.69 (.54, .85)	.63 (.52, .74)	.47 (.29, .60)	.79 (.62, .95)

Supplementary table 10. Shared environmental correlations of BMI for males (upper triangular matrix) and females (lower triangular matrix) from 1 to 19 years of age under ACE model.

	1	2	3	4	5	6	7	8	9	10	11	12	13	14	15	16	17	18	19
19	.42 (-1.00, 1.00)	-1.00 (NA, 1.00)	-.64 (-1.00, 1.00)	NA (NA, NA)	1.00 (-1.00, NA)	NA (NA, NA)	1.00 (-1.00, NA)	1.00 (-1.00, NA)	NA (NA, NA)	.76 (-1.00, 1.00)	1.00 (-1.00, NA)	.93 (-1.00, NA)	1.00 (-1.00, NA)	.41 (-1.00, 1.00)	1.00 (.96, NA)	-1.00 (NA, 1.00)	-1.00 (NA, NA)	.18 (-1.00, 1.00)	
18	-1.00 (NA, 1.00)	-1.00 (NA, 1.00)	1.00 (-1.00, NA)	.40 (NA, 1.00)	.40 (NA, 1.00)	.72 (NA, NA)	1.00 (-1.00, NA)	1.00 (NA, 1.00)	.99 (NA, NA)	1.00 (-1.00, NA)	1.00 (.97, NA)	1.00 (-1.00, NA)	1.00 (-1.00, NA)	1.00 (1.00, NA)	1.00 (-1.00, NA)	1.00 (-1.00, NA)	1.00 (-1.00, NA)		1.00 (NA, NA)
17	-1.00 (NA, 1.00)	-1.00 (NA, 1.00)	.38 (-1.00, 1.00)	1.00 (-1.00, NA)	1.00 (NA, 1.00)	1.00 (-1.00, NA)	1.00 (NA, NA)	1.00 (NA, 1.00)	1.00 (-1.00, NA)	-1.00 (NA, 1.00)	1.00 (NA, NA)	1.00 (-1.00, NA)	.46 (-1.00, 1.00)	1.00 (-1.00, NA)	1.00 (-1.00, NA)	-1.00 NA, 1.00)		.53 (-1.00, NA)	-1.00 (NA, 1.00)
16	-.25 (-1.00, 1.00)	.16 (-1.00, 1.00)	.18 (-1.00, 1.00)	.37 (-1.00, 1.00)	.95 (-1.00, NA)	-.07 (-1.00, 1.00)	.44 (.03, 1.00)	.98 (-1.00, NA)	1.00 (-1.00, NA)	.62 (.46, 1.00)	.59 (-1.00, 1.00)	.86 (-1.00, 1.00)	.78 (.78, NA)	1.00 (-1.00, NA)	1.00 (-1.00, NA)		1.00 (-1.00, NA)	1.00 (-1.00, NA)	-1.00 (NA, 1.00)
15	-.59 (-1.00, 1.00)	.35 (-1.00, 1.00)	.80 (NA, 1.00)	.21 (-1.00, 1.00)	.33 (-.42, 1.00)	.53 (NA, 1.00)	.72 (.63, 1.00)	1.00 (.92, NA)	1.00 (.99, NA)	.81 (-1.00, 1.00)	.82 (-1.00, 1.00)	1.00 (-1.00, NA)	.96 (.96, 1.00)	.93 (-1.00, 1.00)		1.00 (NA, NA)	1.00 (-1.00, NA)	1.00 (NA, NA)	1.00 (-1.00, NA)
14	.15 (-1.00, 1.00)	.49 (.49, 1.00)	.15 (-1.00, 1.00)	.20 (-1.00, 1.00)	1.00 (-1.00, NA)	.27 (-1.00, 1.00)	.52 (.21, .91)	.84 (.84, 1.00)	.52 (-1.00, 1.00)	.79 (-1.00, 1.00)	.69 (.11, .98)	.64 (.64, 1.00)	1.00 (.99, NA)		.98 (-1.00, NA)	1.00 (-1.00, NA)	.99 (-1.00, NA)	.93 (.65, NA)	1.00 (-1.00, NA)
13	-.09 (-.98, NA)	-.01 (-.55,-.01)	.45 (.43, 1.00)	1.00 (-1.00, NA)	.28 (NA, NA)	1.00 (1.00, NA)	1.00 (-1.00, NA)	.80 (NA, NA)	-1.00 (NA, 1.00)	.79 (-.59, NA)	1.00 (1.00, NA)	.91 (.81, NA)		1.00 (-.97, NA)	.69 (-1.00, 1.00)	1.00 (-1.00, NA)	.50 (NA, 1.00)	1.00 (-1.00, NA)	1.00 (.98, NA)

12	.24 (.24, .33)	.12 (-1.00, 1.00)	.15 (.14, .15)	.12 (-.35, .12)	.47 (.47, NA)	1.00 (.45, NA)	.37 (.07, .37)	.95 (.80, 1.00)	-.23 (NA, 1.00)	.80 (.80, .93)	1.00 (.95, NA)		.97 (NA, NA)	.65 (NA, NA)	.96 (-1.00, 1.00)	.30 (NA, 1.00)	1.00 (NA, NA)	1.00 (-1.00, NA)	.99 (-1.00, NA)
11	.27 (-1.00, 1.00)	.20 (-1.00, 1.00)	1.00 (-1.00, NA)	-1.00 (NA, 1.00)	.39 (-1.00, NA)	1.00 (.94, NA)	.42 (.22, .42)	1.00 (-1.00, NA)	.72 (.69, 1.00)	1.00 (-1.00, NA)		1.00 (-1.00, NA)	.45 (-1.00, NA)	.94 (.88, 1.00)	1.00 (-1.00, NA)	.41 (-1.00, 1.00)	1.00 (-1.00, NA)	1.00 (NA, NA)	1.00 (NA, NA)
10	.39 (.23, 1.00)	.59 (-.62, 1.00)	.21 (-1.00, 1.00)	-.03 (-1.00, 1.00)	.15 (-.21, .15)	.93 (-1.00, 1.00)	.27 (-.17, .27)	.77 (.77, 1.00)	-1.00 (NA, 1.00)		1.00 (.99, NA)	1.00 (-1.00, NA)	1.00 (-1.00, NA)	1.00 (1.00, NA)	1.00 (-1.00, NA)	.43 (-.91, 1.00)	1.00 (.85, NA)	1.00 (-1.00, NA)	1.00 (-1.00, NA)
9	.84 (NA, 1.00)	.62 (.31, 1.00)	.27 (-1.00, 1.00)	1.00 (-1.00, NA)	.15 (-1.00, NA)	.44 (-1.00, 1.00)	.12 (-1.00, .53)	1.00 (-1.00, NA)		1.00 (-1.00, NA)	.93 (.93, 1.00)	.93 (-1.00, NA)	-.30 (-1.00, 1.00)	.58 (-.42, .98)	1.00 (-1.00, NA)	-1.00 (NA, 1.00)	.21 (-.17, 1.00)	.91 (.91, 1.00)	NA (NA, NA)
8	.12 (-.27, .16)	.14 (-.59, .35)	-.88 (NA, 1.00)	.12 (.12, 1.00)	.77 (.67, 1.00)	-.01 (-1.00, .44)	1.00 (NA, NA)		1.00 (.82, NA)	.24 (-.32, .24)	1.00 (-1.00, NA)	.68 (.66, 1.00)	-.61 (-1.00, 1.00)	.27 (-1.00, 1.00)	.55 (-1.00, 1.00)	.91 (-1.00, NA)	1.00 (-1.00, NA)	.81 (-1.00, 1.00)	-1.00 (NA, 1.00)
7	.33 (.09, .57)	.23 (.10, NA)	.15 (-.05, .30)	.01 (-1.00, 1.00)	.34 (.30, .52)	.72 (NA, NA)		.97 (.97, 1.00)	.07 (-.39, .07)	.39 (.38, .39)	.76 (.76, 1.00)	.73 (.73, 1.00)	.17 (-1.00, .51)	.84 (.75, 1.00)	.52 (.16, 1.00)	.63 (.51, 1.00)	1.00 (-1.00, NA)	1.00 (-1.00, NA)	1.00 (NA, NA)
6	.45 (.45, .70)	.08 (-1.00, 1.00)	.74 (-1.00, 1.00)	.04 (NA, NA)	.46 (NA, 1.00)		.52 (-1.00, 1.00)	.32 (-1.00, 1.00)	.26 (-1.00, 1.00)	-1.00 (NA, 1.00)	1.00 (-1.00, NA)	-.00 (NA, 1.00)	-1.00 (NA, 1.00)	1.00 (-1.00, NA)	1.00 (-1.00, NA)	.99 (-1.00, NA)	-1.00 (NA, 1.00)	.67 (NA, NA)	NA (NA, NA)
5	.35 (NA, .39)	.36 (NA, .45)	.70 (-.83, NA)	1.00 (-1.00, NA)		-.19 (NA, NA)	.60 (.49, .84)	.12 (NA, .12)	-1.00 (NA, NA)	.85 (-1.00, 1.00)	.73 (.73, NA)	.26 (.26, 1.00)	.86 (NA, 1.00)	1.00(-1.00, NA)	1.00 (-1.00, NA)	.09 (-1.00, 1.00)	-1.00 (NA, 1.00)	1.00 (.78, NA)	1.00 (-1.00, NA)
4	.66 (.66, 1.00)	.34 (.04, .35)	.32 (.20, .42)		.71 (.58, NA)	.30 (NA, NA)	.11 (-.12, .11)	.70 (NA, 1.00)	.20 (-.10, .20)	.35 (-1.00, 1.00)	.19 (.13, .22)	.23 (-1.00, 1.00)	.12 (-1.00, .12)	1.00 (-	.35 (-1.00, 1.00)	.02 (-	1.00 (.96, NA)	.31 (.31, 1.00)	NA (NA, NA)

														1.00, NA)		1.00, 1.00)			
3	.48 (.35, .59)	.60 (NA, .71)		.26 (.09, .39)	.51 (-1.00, .70)	-.94 (-.00, 1.00)	.24 (.05, .38)	-.99 (NA, NA)	-1.00 (NA,-.80)	.20 (.20, .20)	.13 (-.17, .13)	-1.00 (NA, 1.00)	.06 (-.34, .06)	1.00 (NA, NA)	-.10 (-.00, 1.00)	.29 (.00, 1.00)	1.00 (-1.00, NA)	1.00 (-1.00, NA)	1.00 (-1.00, NA)
2	.34 (.15, NA)		.83 (NA, 1.00)	.34 (.34, 1.00)	.35 (.10, NA)	1.00 (-1.00, NA)	.33 (NA, .33)	.05 (-1.00, 1.00)	1.00 (-1.00, NA)	.53 (-1.00, 1.00)	1.00 (.97, NA)	.30 (.24, .55)	-1.00 (NA,-1.00)	.42 (.42, 1.00)	.38 (-1.00, 1.00)	1.00 (-1.00, NA)	-1.00 (NA, 1.00)	1.00 (-1.00, NA)	-1.00 (NA, 1.00)
1		.34 (.14, .49)	.52 (NA, .60)	.48 (.48, 1.00)	.07 (-.51, NA)	1.00 (-1.00, NA)	.24 (-.07, 1.00)	.20 (-1.00, 1.00)	1.00 (NA, NA)	.08 (-1.00, 1.00)	1.00 (-1.00, NA)	.10 (-1.00, 1.00)	-.40 (-1.00, 1.00)	.98 (NA, NA)	-1.00 (NA, 1.00)	1.00 (.57, NA)	-1.00 (-1.00, NA)	1.00 (-1.00, NA)	1.00 (-1.00, NA)

Supplementary table 11. Shared environmental correlations of height for males (upper triangular matrix) and females (lower triangular matrix) from 1 to 19 years of age under ACE model.

	1	2	3	4	5	6	7	8	9	10	11	12	13	14	15	16	17	18	19
19	.61 (-1.00, 1.00)	.72 (.28, 1.00)	.77 (.32, 1.00)	NA (NA, NA)	.50 (-.19, .80)	NA (NA, NA)	.42 (-1.00, .83)	1.00 (-1.00, NA)	.83 (-1.00, 1.00)	.68 (-1.00, 1.00)	.82 (.55, 1.00)	1.00 (-1.00, NA)	.50 (-1.00, 1.00)	1.00 (NA, NA)	-1.00 (NA, NA)	1.00 (-.95, NA)	1.00 (-1.00, NA)	.94 (NA, 1.00)	
18	1.00 (NA, NA)	1.00 (NA, NA)	1.00 (NA, NA)	.55 (NA, NA)	.70 (-.18, 1.00)	1.00 (NA, NA)	1.00 (-1.00, NA)	.51 (-1.00, 1.00)	1.00 (-1.00, NA)	1.00 (-1.00, NA)	1.00 (NA, NA)	1.00 (-1.00, NA)	1.00 (-1.00, NA)	1.00 (NA, NA)	-1.00 (NA, 1.00)	1.00 (NA, NA)	1.00 (-1.00, NA)		.97 (-1.00, 1.00)
17	.62 (-1.00, 1.00)	1.00 (NA, NA)	1.00 (.99, NA)	1.00 (-1.00, NA)	.77 (-.10, 1.00)	1.00 (NA, NA)	1.00 (-1.00, NA)	1.00 (NA, NA)	.89 (-1.00, NA)	.74 (-.26, NA)	1.00 (.91, NA)	1.00 (NA, NA)	1.00 (-1.00, NA)	-1.00 (NA, 1.00)	1.00 (NA, NA)	1.00 (-1.00, NA)		1.00 (NA, NA)	.98 (.95, 1.00)
16	1.00 (.50, NA)	.94 (.94, NA)	1.00 (.82, NA)	1.00 (.75, NA)	1.00 (-1.00, NA)	.99 (-1.00, NA)	1.00 (NA, NA)	-.19 (-1.00, 1.00)	1.00 (NA, NA)	1.00 (.91, NA)	1.00 (-1.00, NA)	1.00 (-1.00, NA)	.71 (-.13, 1.00)	-1.00 (NA, 1.00)	1.00 (NA, NA)		1.00 (.99, NA)	1.00 (NA, NA)	1.00 (NA, NA)
15	1.00 (.62, NA)	1.00 (-1.00, NA)	.77 (-1.00, 1.00)	1.00 (NA, NA)	.91 (.91, NA)	1.00 (-1.00, NA)	.70 (-1.00, 1.00)	-.91 (-1.00, 1.00)	1.00 (NA, NA)	1.00 (-1.00, NA)	.76 (-1.00, NA)	1.00 (-1.00, NA)	.93 (-1.00, NA)	.86 (-1.00, NA)		1.00 (NA, NA)	1.00 (NA, NA)	1.00 (NA, NA)	1.00 (NA, NA)
14	1.00 (.52, NA)	1.00 (1.00, NA)	1.00 (-1.00, NA)	1.00 (-1.00, NA)	1.00 (-1.00, NA)	-1.00 (NA, 1.00)	1.00 (-1.00, NA)	1.00 (-1.00, NA)	-1.00 (NA, 1.00)	1.00 (-1.00, NA)	.95 (.74, NA)	.38 (-1.00, 1.00)	1.00 (-1.00, NA)		.80 (NA, 1.00)	.92 (.80, 1.00)	.97 (.83, NA)	1.00 (.91, NA)	.70 (-1.00, 1.00)
13	.83 (.83, 1.00)	.56 (.56, 1.00)	.75 (.48, 1.00)	1.00 (.97, NA)	.67 (.67, 1.00)	1.00 (1.00, NA)	.71 (-1.00, 1.00)	.84 (NA, 1.00)	.49 (-1.00, 1.00)	.75 (-1.00, 1.00)	1.00 (1.00, NA)	NA (NA, NA)		.66 (-1.00, NA)	.91 (.91, NA)	1.00 (NA, NA)	1.00 (NA, NA)	1.00 (-1.00, NA)	.77 (-1.00, 1.00)

12	.82 (.58, 1.00)	.59 (.59, .83)	.53 (.53, .81)	1.00 (.99, NA)	.99 (.99, NA)	1.00 (- 1.00, NA)	.59 (.59, 1.00)	.93 (- 1.00, NA)	-.45 (- 1.00, 1.00)	.62 (- 1.00, .80)	1.00 (- 1.00, NA)		NA (NA, NA)	.50 (- .51, .99)	.74 (- 1.00, 1.00)	1.00 (.70, NA)	1.00 (- 1.00, NA)	.55 (- 1.00, 1.00)	.72 (.23, 1.00)
11	.51 (- 1.00, 1.00)	.48 (.35, .64)	.33 (- 1.00, 1.00)	.52 (NA, 1.00)	.94 (NA, NA)	.61 (- 1.00, 1.00)	.82 (.82, .96)	1.00 (.83, NA)	.77 (.52, .87)	1.00 (.96, NA)		1.00 (- 1.00, NA)	1.00 (- 1.00, NA)	.70 (.09, .93)	.67 (.67, NA)	.56 (.08, NA)	.98 (.67, NA)	1.00 (NA, NA)	-.20 (- 1.00, 1.00)
10	.67 (.54, .82)	.55 (.47, .57)	.59 (.59, .77)	.49 (.48, .67)	.50 (NA, .69)	.81 (- 1.00, 1.00)	.71 (.67, .78)	.67 (NA, .79)	1.00 (- 1.00, NA)		1.00 (.62, NA)	1.00 (1.00, NA)	1.00 (NA, NA)	1.00 (.72, NA)	1.00 (NA, NA)	.66 (- 1.00, 1.00)	.70(.70, NA)	.99 (- 1.00, NA)	.46 (- 1.00, .86)
9	.56 (- 1.00, 1.00)	.31 (- .09, .61)	.41 (NA, .63)	.45 (- 1.00, 1.00)	.67 (NA, 1.00)	.18 (- 1.00, NA)	.65 (.50, .74)	.90 (- .41, NA)		.80 (- 1.00, 1.00)	.81 (- .58, 1.00)	1.00 (- 1.00, NA)	.42 (- .75, .80)	-.00 (- 1.00, .58)	.24 (- 1.00, .92)	.32 (- 1.00, 1.00)	.71 (- 1.00, 1.00)	- 1.00 (NA, 1.00)	.44 (- 1.00, 1.00)
8	.48 (.45, .72)	.47 (.40, .65)	.27 (- .30, .58)	.42 (- .03, .62)	.61 (.27, .75)	.72 (.47, .84)	.61(- 1.00, 1.00)		.89 (.88, 1.00)	.71 (NA, NA)	1.00 (.97, NA)	.53 (- 1.00, 1.00)	.85 (.85, 1.00)	.01 (- 1.00, 1.00)	.86 (- 1.00, 1.00)	.24 (- 1.00, 1.00)	.88 (.56, NA)	1.00 (.70, NA)	.81 (.44, 1.00)
7	.63 (.52, .73)	.64 (.47, .75)	.37 (NA, .51)	.35 (.12, .49)	.69 (NA, .77)	.76 (- .49, 1.00)		.82 (NA, NA)	.58 (.31, .71)	.69 (.56, .78)	.75 (.75, 1.00)	.81 (.63, 1.00)	1.00 (- 1.00, NA)	.75 (.75, 1.00)	.88 (.66, 1.00)	.47 (.47, .81)	.93 (- 1.00, NA)	.94 (- 1.00, NA)	1.00 (- 1.00, NA)
6	.39 (.39, 1.00)	.57 (.43, .77)	.63 (.16, .84)	.67 (NA, NA)	.80 (NA, NA)		.65 (- .62, .89)	.53 (- .08, .70)	-.17 (- 1.00, .46)	.23 (- .65, .72)	- 1.00 (NA, 1.00)	.65 (- 1.00, 1.00)	.69 (- 1.00, 1.00)	.85 (.85, NA)	.96 (- 1.00, NA)	.74 (NA, 1.00)	.87 (- 1.00, NA)	.61 (- 1.00, 1.00)	NA (NA, NA)
5	.49 (NA, .59)	.48 (NA, .61)	.6 1(.42, .72)	.62 (.35, .75)		.96 (NA, 1.00)	.73 (.62, .84)	.59 (.59, .79)	.39 (- 1.00, 1.00)	.60 (.51, .61)	.81 (.81, 1.00)	.99 (NA, NA)	.73 (- 1.00, 1.00)	.68 (NA, 1.00)	.87 (.53, 1.00)	1.00 (- 1.00, NA)	.85 (- 1.00, 1.00)	1.00 (- 1.00, NA)	1.00 (- 1.00, NA)
4	.39 (NA, .54)	.53 (.35, .64)	.36 (.17, .49)		.48 (- .22, .70)	.64 (- 1.00, NA)	.22(- .26, .45)	.72 (NA, NA)	.14 (- 1.00, 1.00)	1.00 (1.00, NA)	.36 (-	.51 (- 1.00, 1.00)	1.00 (-	.09 (-	.51 (.42, .82)	.51 (-	.79 (-	.65 (-	NA (NA, NA)

											1.00, 1.00)		1.00, NA)	1.00, 1.00)		1.00, 1.00)	1.00, 1.00)	1.00, NA)	
3	.58 (.56, .65)	.74 (NA, NA)		.45 (.28, .57)	.65 (.49, .75)	.69 (.56, NA)	.45 (NA, .57)	.33 (-1.00, .61)	.54 (.54, 1.00)	.67 (.54, 1.00)	.37 (-1.00, 1.00)	.89 (.89, 1.00)	.77 (.77, 1.00)	.65 (.63, 1.00)	.77 (.56, .97)	.15 (-.18, .44)	1.00 (-1.00, NA)	.59 (-.95, 1.00)	.87 (NA, NA)
2	.62 (.54, .69)		.80 (.80, .87)	.54 (.36, .66)	.55 (.50, .66)	.96 (-1.00, NA)	.60(NA, .76)	.77 (.77, 1.00)	.96 (-1.00, NA)	.40 (.09, .62)	1.00 (-1.00, NA)	.56 (-1.00, 1.00)	.84 (.84, 1.00)	.72 (.18, 1.00)	.80 (-1.00, 1.00)	1.00 (-1.00, NA)	.89 (.89, NA)	1.00 (-1.00, NA)	.79 (NA, 1.00)
1		.60 (.59, .67)	.53 (.51, .61)	.45 (.41, .58)	.47 (.44, .59)	.27 (-1.00, NA)	.46(NA, NA)	.42 (-1.00, 1.00)	.00 (-1.00, 1.00)	.48 (.32, .62)	1.00 (-1.00, NA)	.67 (.44, 1.00)	.86 (.45, 1.00)	.87 (.61, 1.00)	.79 (.79, 1.00)	.89 (.89, NA)	1.00 (.96, NA)	1.00 (NA, NA)	.56 (-1.00, 1.00)

Supplementary table 12. Unique environmental correlations of BMI for males (upper triangular matrix) and females (lower triangular matrix) from 1 to 19 years of age under ACE model.

	1	2	3	4	5	6	7	8	9	10	11	12	13	14	15	16	17	18	19
19	-.05 (NA, .18)	.41 (.09, .65)	.16 (-.14, .44)	NA (NA, NA)	-.11 (-.38, .19)	NA (NA, NA)	.05 (NA, NA)	.28 (-.01, .54)	NA (NA, NA)	.49 (.26, .67)	.07 (NA, NA)	.00 (-.21, .22)	.18 (-.07, .40)	.15 (-.09, .38)	.73 (.57, .83)	.30 (NA, .41)	.41 (NA, .51)	.34 (.17, .49)	
18	.20 (.06, NA)	.08 (-.09, .25)	.21 (.04, .38)	.29 (NA, NA)	.20 (NA, NA)	.49 (NA, NA)	.16 (.00, .32)	.21 (.01, .39)	.37 (NA, NA)	.34 (.21, .46)	.28 (.12, .42)	.14 (.02, .26)	.40 (.23, .55)	.28 (.17, .39)	.48 (.31, NA)	.39 (.27, .49)	.48 (.35, .59)		.74 (.64, .82)
17	.06 (-.08, .21)	.27 (.10, .42)	.20 (.05, .33)	.16 (-.03, .35)	.03 (-.19, .25)	.44 (.14, .67)	.10 (-.02, .23)	.28 (.06, .47)	.18 (.03, .33)	-.03 (-.18, .12)	.26 (.14, .37)	.33 (.22, .43)	.07 (-.10, .23)	.28 (.17, .38)	.27 (.13, .40)	.55 (.48, .61)		.57 (.47, .65)	.38 (NA, NA)
16	.22 (.06, .36)	.30 (.14, .46)	.16 (.02, .29)	.27 (.12, .42)	.53 (.33, .68)	.32 (NA, NA)	.40 (.30, .50)	.20 (.04, .35)	.47 (NA, NA)	.45 (.35, .54)	.37 (.21, .50)	.50 (.41, .58)	.22 (NA, .35)	.54 (.46, .62)	.49 (.27, NA)		.62 (.57, .66)	.48 (.38, .57)	.37 (.29, .46)
15	.13 (.01, .25)	.20 (.06, .33)	.10 (-.04, .23)	.07 (-.08, .22)	.24 (.07, .41)	.21 (.00, .40)	.27 (.17, .37)	.38 (.23, .52)	.36 (.21, .49)	.25 (.13, .35)	.36 (.24, .48)	.47 (.39, .55)	.47(.35, .57)	.71 (NA, .82)		.62 (.40, NA)	.35 (.23, .47)	.44 (.29, NA)	.40 (.24, .55)
14	.19 (.07, .31)	.28 (.15, .40)	.09 (-.02, .21)	.16 (.03, .28)	.39 (.24, .52)	.38 (NA, NA)	.26 (.16, .36)	.44 (NA, NA)	.49 (.38, .59)	.40 (.30, .49)	.41 (.33, .48)	.50 (.44, .56)	.63 (NA, NA)		.71 (NA, NA)	.42 (.33, .51)	.35 (.26, .44)	.44 (.35, .52)	.21 (-.01, .42)
13	.17 (.06, .28)	.25 (.13, .36)	.24 (.11, .37)	.26 (.10, .40)	.23 (.09, .35)	.44 (.27, .59)	.38 (.28, .48)	.51(NA, NA)	.38 (.26, .49)	.44 (.33, .53)	.61 (.51, .69)	.70 (NA, NA)		.22 (NA, .35)	.47 (.34, .58)	.21 (.07, NA)	.45 (.30, .57)	.18 (-.12, .44)	.26 (.06, .44)
12	.23 (.16, .29)	.19 (NA, .27)	.27 (.20, .34)	.26 (.16, .35)	.37 (NA, NA)	.37 (.24, .49)	.37 (.31, .43)	.57 (.50, .63)	.62 (NA, NA)	.61 (.56, .65)	.70 (.64, .74)		.12 (NA, NA)	.38 (.32, .44)	.45(.37, .54)	.37 (.27, .46)	.26 (.16, .36)	.42 (.32, .51)	.28 (.12, .42)
11	.18 (.09, .27)	.24 (.12, .34)	.27 (.19, .35)	.25 (.16, .33)	.60 (NA, NA)	.47 (.36, NA)	.43 (.37, .48)	.64 (.58, .69)	.57 (.51, .62)	.58 (.51, .64)		.62 (.57, .67)	.49 (.39, .58)	.34 (.26, .41)	.42(.28, .54)	.31 (.18, .44)	.39 (.28, .48)	.23 (.07, .38)	.19 (NA, NA)

10	.26 (.20, .32)	.30 (.23, .37)	.36 (.30, .42)	.32 (.24, .40)	.44 (.36, .51)	.51 (.42, .59)	.46 (.41, .50)	.62 (.57, .67)	.65 (.59, .70)		.72 (.67, .76)	.59 (.55, .63)	.56 (.47, .65)	.52 (.43, .60)	.34(.23, .44)	.39 (.28, .49)	.37 (.25, .48)	.28 (.12, .43)	.27 (.08, .44)
9	.21 (.12, .29)	.36 (.26, .45)	.40 (.31, .48)	.29 (.19, .39)	.46 (.36, .55)	.52 (.43, .61)	.50 (.44, .56)	.72 (.67, .76)		.73 (.68, .77)	.59 (.54, .64)	.46 (NA, NA)	.44 (.30, .55)	.41 (.30, .51)	.56(.40, .68)	.01 (NA, NA)	.29 (.13, .42)	.51 (NA, NA)	NA (NA, NA)
8	.27 (.20, .34)	.29 (.21, .37)	.41 (.33, .48)	.38 (.28, .47)	.52 (.43, .60)	.51 (NA, .59)	.64 (NA, .69)		.75 (.70, .79)	.65 (.60, .69)	.66 (.60, .71)	.43 (.36, .50)	.15 (NA, NA)	.29 (NA, .42)	.52(.35, .65)	.36 (NA, .50)	.46 (.26, .62)	.42 (.15, .63)	.47 (.28, .63)
7	.31 (.25, .36)	.33 (.26, .39)	.34 (.28, .39)	.32 (.26, .39)	.46 (.39, .52)	.62 (NA, .69)		.60 (NA, NA)	.45 (.39, .51)	.41 (.36, .45)	.42 .37, .48)	.38 (.32, .43)	.43 (.33, .53)	.30 (.22, .39)	.32(.21, .41)	.20 (.09, .30)	.19 (.07, .30)	.37 (.16, .55)	.19 (NA, NA)
6	.27 (.17, .35)	.34 (.24, .43)	.42 (NA, NA)	.52 (NA, NA)	.53 (NA, NA)		.51 (NA, NA)	.58 (NA, .65)	.55 (.46, .63)	.47 (.38, .55)	.47 (.36, NA)	.49 (.36, .59)	.39 (.18, .58)	.51 (NA, NA)	.21(- .11, .50)	.48 (NA, NA)	.64 (.21, .86)	-.35 (NA, NA)	NA (NA, NA)
5	.38 (NA, NA)	.44 (.38, .50)	.49 (.43, .54)	.61 (.54, NA)		.63 (.53, .71)	.62 (.56, .66)	.41 (.31, .50)	.49 (.39, .59)	.43 (.36, .50)	.57 (NA, NA)	.28 (NA, NA)	.34 (.21, .46)	.27 (.13, .52)	.25 (.09, .40)	.49 (.30, .64)	.16 (.03, .33)	.29 (NA, NA)	.36 (.09, .58)
4	.41 (.34, .48)	.48 (.41, .54)	.41 (.36, .47)		.66 (.60, NA)	.43 (NA, NA)	.31 (.24, .37)	.40 (.30, .49)	.44 (.35, .52)	.29 (.21, .36)	.32 (.24, .39)	.25 (.16, .34)	.51 (.38, .63)	.23 (.11, .34)	.23 (.04, .40)	.19 (.05, .33)	.33 (.16, .48)	-.35 (NA, NA)	NA (NA, NA)
3	.41 (.37, .45)	.42 (.37, .46)		.41 (.35, .46)	.54 (.49, .59)	.51 (NA, NA)	.45 (.40, .49)	.53 (.47, .59)	.43 (.34, .50)	.33 (.27, .39)	.38 (.31, .44)	.24 (.18, .31)	.28 (.15, .40)	.18 (.08, .28)	.22 (.09, .34)	.13 (- .00, .25)	.19 (.07, .31)	.19 (- 03, .39)	.27 (.04, .47)
2	.44 (.40, .48)		.58 (.54, .61)	.50 (.43, .57)	.49 (.43, .54)	.45 (.35, .54)	.44 (.38, .49)	.40 (.32, .47)	.40 (.31, .49)	.32 (.26, .39)	.37 (.27, .46)	.26 (NA, .33)	.26 (.13, .38)	.14 (.02, .26)	.19 (.05, .32)	.37(.19, .52)	.11 (- .04, .28)	.14 (- .13, .38)	.33 (.08, .54)
1		.52 (.48, .55)	.51 (.48, .55)	.50 (.43, .56)	.33 (NA, NA)	.34 (.24, .42)	.35 (.30, .40)	.46 (.40, .52)	.39 (.31, .47)	.29 (.24, .35)	.33 (.25, .41)	.22 (.16, .28)	.23 (.11, .33)	.28 (.17, .38)	.02 (- .09, .14)	.24(.10, .38)	.17 (.04, .29)	-.02 (NA, NA)	.27 (.11, .42)

Supplementary table 13. Unique environmental correlations of height for males (upper triangular matrix) and females (lower triangular matrix) from 1 to 19 years of age under ACE model.

	1	2	3	4	5	6	7	8	9	10	11	12	13	14	15	16	17	18	19
19	.29 (.07, .49)	.10 (-.25, .42)	.44 (.18, .65)	NA (NA, NA)	.68 (NA, NA)	NA (NA, NA)	.42 (.20, .61)	.06 (-.23, .35)	.61 (.36, .78)	.39 (.14, .59)	.63 (NA, NA)	.04 (-.18, .26)	.28 (.05, .47)	.26 (.02, .47)	.61 (.41, .75)	.51 (.42, .60)	.55 (.46, .63)	.79 (NA, NA)	
18	.42 (.29, .53)	.40 (.25, .54)	.44 (.29, .58)	.48 (.32, .61)	.38 (.17, .56)	.52 (NA, NA)	.44 (.30, .56)	.41 (NA, NA)	.52 (NA, NA)	.60 (.49, .68)	.42 (.28, .55)	.46 (.36, .55)	.49(.34, .62)	.41 (.31, .50)	.19 (-.00, .37)	.66 (.57, .73)	.70 (.61, .77)		.91 (NA, NA)
17	.40 (.27, .51)	.25 (.09, .41)	.29 (.15, .41)	.40 (.22, .55)	.54 (.36, .68)	.28 (-.04, NA)	.49 (.39, .58)	.42 (.22, .59)	.49 (.36, .60)	.50 (NA, NA)	.55 (.45, .63)	.35 (.24, .44)	.43 (.29, .56)	.31 (.20, .41)	.55 (.43, .64)	.72 (.67, .77)		.73 (.67, .78)	.83 (.79, .86)
16	.30 (.15, .43)	.46 (.31, .59)	.18 (.03, .31)	.08 (-.08, .25)	.44 (.21, .62)	.46 (NA, NA)	.34 (.22, .45)	.32 (.17, .46)	.67 (.46, .80)	.55 (.46, .63)	.32 (NA, .47)	.40 (.30, .49)	.40 (.26, .52)	.54 (.44, .62)	.58 (NA, NA)		.76 (.73, .79)	.79 (.73, .83)	.71 (.66, .76)
15	.29 (.17, .40)	.37 (.24, .48)	.37 (.25, .48)	.46 (.33, .58)	.46 (.30, .59)	.36 (.16, .52)	.36 (.26, .45)	.52 (.39, .64)	.69 (.59, .76)	.41 (.31, .50)	.26 (.14, NA)	.44 (.35, .51)	.58 (.47, .66)	.80 (NA, NA)		.49 (NA, .66)	.52 (.43, .61)	.73 (.63, .81)	.67 (NA, .77)
14	.17 (.05, .29)	.32 (.19, .44)	.28 (.18, .39)	.29 (.17, .40)	.27 (.12, .41)	.46 (.28, .61)	.35 (.25, .43)	.47 (.34, .58)	.54 (.44, .63)	.55 (.47, .63)	.40 (.32, .47)	.47 (.41, .53)	.55 (.42, .66)		.88 (NA, NA)	.56 (.47, .63)	.57 (.50, .64)	.67 (.60, .73)	.52 (.34, .66)
13	.29 (.18, .39)	.35 (.24, .46)	.51 (.40, .60)	.29 (.14, .43)	.46 (.34, .57)	.44 (.27, .58)	.57 (.49, .64)	.57 (.47, .65)	.45 (.33, .55)	.65 (.58, .72)	.79 (.74, .84)	NA (NA, NA)		.45 (.32, .56)	.70 (.62, .77)	.32 (.17, .45)	.41 (.26, .54)	.65 (.46, .79)	.40 (.22, .56)
12	.32 (.26, .38)	.38 (.30, .45)	.40 (.33, .46)	.46 (.37, .54)	.50 (.43, .57)	.55 (.44, .64)	.47 (.41, .52)	.65 (.60, .70)	.72 (.66, .77)	.66 (.62, .69)	.76 (.72, .80)		NA (NA, NA)	.55 (.49, .60)	.60 (.53, .66)	.27 (.17, .36)	.33 (.23, .43)	.52 (.43, .59)	.32 (.17, .46)
11	.29 (.20, .37)	.23 (.12, .34)	.30 (.23, .38)	.44 (.37, .51)	.60 (.51, .68)	.60 (.51, .68)	.61 (.57, .66)	.75 (.70, .79)	.77 (.74, .80)	.71 (.66, .76)		.67 (.62, .72)	.70 (.63, .77)	.54 (.48, .59)	.51 (.39, NA)	.31 (NA, NA)	.45 (.36, .54)	.52 (.38, .63)	.65 (NA, NA)
10	.46 (.41, .51)	.50 (.44, .55)	.54 (.49, .59)	.51 (.44, .57)	.66 (.60, .71)	.74(.68, .79)	.59 (.55, .63)	.75 (.72, .79)	.77 (.73, .81)		.79 (.76, .82)	.75 (.72, .78)	.67 (.59, .74)	.45 (.35, .53)	.62 (.54, .69)	.53 (.44, .61)	.47 (NA, NA)	.56 (.44, .67)	.40 (.23, .55)

9	.44 (.36, .51)	.37 (.27, .46)	.60 (.53, .66)	.50 (.41, .57)	.55 (.46, .63)	.71 (.64, .77)	.72 (.69, .76)	.82 (.78, .85)		.77 (.73, .80)	.73 (.69, .77)	.70 (.65, .76)	.62 (.52, .70)	.52 (.42, .60)	.53 (.37, .65)	.64 (.37, .81)	.51 (.39, .62)	.82 (NA, .93)	.58 (.29, .78)
8	.43 (.36, .49)	.45 (.37, .52)	.55 (.48, .60)	.56 (.47, .63)	.67 (.60, .73)	.75 (.70, .80)	.84 (NA, .87)		.82 (.79, .85)	.78 (.74, .81)	.73 (.68, .77)	.68 (.63, .73)	.58 (.48, .66)	.64 (.53, .72)	.57 (.41, .69)	.67 (.56, .76)	.63 (.46, .75)	.70 (.52, NA)	.52 (.34, .67)
7	.50 (.45, .54)	.53 (.48, .58)	.47 (.42, .51)	.47 (.41, .52)	.72 (.68, .76)	.72 (.67, .77)		.82 (.78, .84)	.74 (.70, .77)	.68 (.65, .71)	.61 (.56, .65)	.61 (.56, .64)	.55 (.45, .63)	.54 (.47, .60)	.65 (.58, .71)	.45 (.36, .54)	.45 (.35, .53)	.59 (.42, .72)	.45 (.27, .60)
6	.43 (.35, .50)	.52 (.44, .60)	.50 (.42, .58)	.68 (NA, NA)	.73 (NA, NA)		.75 (.70, .79)	.71 (.65, .76)	.63 (.55, .70)	.62 (.55, .69)	.57 (.47, .65)	.52 (.40, .62)	.70 (.57, .80)	.71 (.59, .80)	.47 (.18, .68)	.78 (NA, NA)	.82 (.58, NA)	.65 (NA, NA)	NA (NA, NA)
5	.43 (.37, .48)	.51 (.45, .56)	.61 (.56, .65)	.68 (.62, .73)		.85 (NA, NA)	.73 (.70, .77)	.70 (.64, .76)	.61 (.52, .68)	.57 (.51, .62)	.66 (.58, .73)	.48 (.40, .55)	.35 (.21, .46)	.42 (.29, .54)	.45 (.31, .57)	.66 (.52, .77)	.33 (.15, .49)	.51 (.29, .67)	.54 (NA, NA)
4	.44 (.37, .50)	.54 (.48, .60)	.58 (.53, .62)		.76 (.71, .81)	.60 (NA, NA)	.49 (.44, .54)	.58 (.50, .65)	.46 (.38, .54)	.43 (.36, .50)	.48 (.41, .54)	.30 (.21, .39)	.47 (.32, .59)	.35 (.24, .45)	.50 (.34, .63)	.51 (.40, .61)	.30 (.13, .46)	.83 (.47, .95)	NA (NA, NA)
3	.50 (.46, .54)	.56 (.52, .59)		.57 (.52, .61)	.72 (.68, .75)	.65 (.58, .71)	.56 (.52, .59)	.67 (.62, .71)	.55 (.48, .62)	.55 (.50, .59)	.42 (.36, .48)	.41 (.35, .47)	.47 (.36, .57)	.41 (.32, .49)	.62 (.53, .69)	.15 (.02, .27)	.42 (.31, .51)	.66 (.52, .77)	.28 (.06, .48)
2	.54 (.51, .57)		.66 (.63, .68)	.58 (.52, .64)	.61 (.56, .65)	.57 (.49, .65)	.64 (.60, .68)	.54 (.48, .60)	.49 (.41, .57)	.46 (.40, .52)	.43 (.34, .52)	.41 (.34, .48)	.43 (.31, .54)	.49 (.39, .58)	.45 (.32, .55)	.50 (.35, .63)	.44 (.31, .56)	.70 (.54, .81)	.29 (.04, .51)
1		.52 (.48, .55)	.51 (.47, .54)	.46 (.39, .53)	.44 (.39, .49)	.33 (.24, .42)	.46 (.41, .51)	.39 (.32, .45)	.37 (.28, .45)	.42 (.38, .47)	.25 (.16, .33)	.31 (.25, .37)	.36 (.26, .46)	.39 (.29, .48)	.47 (.38, .56)	.43 (.30, .55)	.45 (.35, .55)	.58 (.46, .68)	.26 (.09, .41)
